# IL-21/IL-21R signaling renders acute myeloid leukemia stem cells more susceptible to cytarabine treatment and CAR T cell therapy

**DOI:** 10.1016/j.xcrm.2024.101826

**Published:** 2024-11-12

**Authors:** Viviana Rubino, Michelle Hüppi, Sabine Höpner, Luigi Tortola, Noah Schnüriger, Hugo Legenne, Lea Taylor, Svenja Voggensperger, Irene Keller, Remy Bruggman, Marie-Noëlle Kronig, Ulrike Bacher, Manfred Kopf, Adrian F. Ochsenbein, Carsten Riether

**Affiliations:** 1 Department of Medical Oncology, Inselspital, Bern University Hospital, University of Bern, Bern, Switzerland; 2 Department for BioMedical Research (DBMR), University of Bern, Bern, Switzerland; 3 Graduate School of Cellular and Biomedical Sciences, University of Bern, Bern, Switzerland; 4 Institute for Molecular Health Sciences, Department of Biology, ETH Zurich, Zurich, Switzerland; 5 Interfaculty Bioinformatics Unit and SIB Swiss Institute of Bioinformatics, University of Bern, Bern, Switzerland; 6 Department of Hematology and Central Hematology Laboratory, Inselspital, Bern University Hospital, University of Bern, Bern, Switzerland

**Keywords:** AML, IL-21, leukemia stem cell, self-renewal, stemness, p38 MAPK, CD70-targeting CAR T cells, cytarabine

## Abstract

Self-renewal programs in leukemia stem cells (LSCs) predict poor prognosis in patients with acute myeloid leukemia (AML). We identify CD4^+^ T cell-derived interleukin (IL)-21 as an important negative regulator of self-renewal of LSCs. IL-21/IL-21R signaling favors asymmetric cell division and differentiation in LSCs through the activation of p38-MAPK signaling, resulting in reduced LSC numbers and significantly prolonged survival in murine AML models. In human AML, serum IL-21 at diagnosis is identified as an independent positive prognostic biomarker for outcome and correlates with improved survival and higher complete remission rates in patients that underwent high-dose chemotherapy. IL-21 treatment inhibits primary LSC function and enhances the effect of cytarabine and CD70 CAR T cell treatment on LSCs *in vitro*. Low-dose IL-21 treatment prolongs the survival of AML mice in syngeneic and xenograft experiments. Therefore, promoting IL-21/IL-21R signaling on LSCs may be an approach to reduce stemness and increase differentiation in AML.

## Highlights

• CD4^+^ T cell-derived IL-21 reduces stemness and promotes the differentiation of AML LSCs
• IL-21 reduces LSC function by activating ROS and p38-MAPK signaling
• Low-dose IL-21 treatment prolongs the survival of MLL-AF9 and PDX AML mice
• IL-21 treatment synergized with ARA-C and CAR T cell treatment

## Graphical abstract

**Figure:**
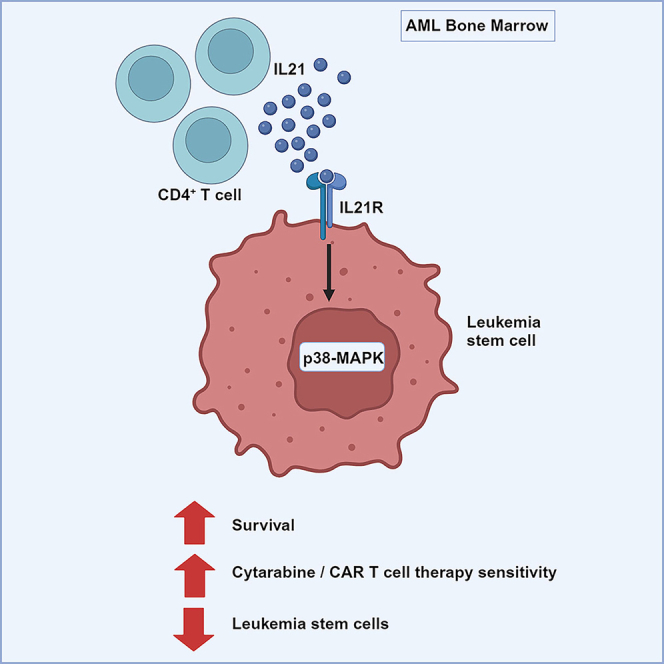

## Teaser

Rubino et al. show that CD4^+^ T cell-derived IL-21 promotes LSC differentiation by activating MAPK signaling and that IL-21 treatment synergistically reduces LSCs in combination with chemotherapy and CAR T cell treatment. These results provide a rationale for the use of IL-21 in the treatment of patients with AML.

## Introduction

Acute myeloid leukemia (AML) is an aggressive myeloid malignancy with poor prognosis.^1^^,^^2^^,^^3^ The standard of care for young and fit patients with AML consists of intensive chemotherapy, followed by consolidation with chemotherapy or allogeneic hematopoietic stem cell (HSCs) transplantation.^4^^,^^5^ Recently, targeted therapies were introduced and have improved prognosis for distinct genetic subgroups.^6^ For patients who cannot tolerate intensive chemotherapy, hypomethylating agents combined with the BCL-2 inhibitor venetoclax became the standard of (palliative) AML therapy.^7^ Despite these developments, most of them will ultimately relapse often with a refractory disease.^5^

Leukemia stem cells (LSCs) are the initiator and driver of the disease and the major cause of relapse.^8^^,^^9^^,^^10^^,^^11^ LSCs rely on interactions with the bone marrow (BM) microenvironment in which they reside for their regulation and maintenance.^12^^,^^13^^,^^14^^,^^15^

Interleukin-21 (IL-21)/IL-21R signaling is variously involved in immune responses. The IL-21R is expressed on several lymphoid and myeloid cell populations and, upon ligation, mainly signals via Janus tyrosine kinases and signal transducers and activators of transcription and, to a lesser extent, also via phosphoinositol 3-kinase/Akt and mitogen-activated protein kinase (MAPK) pathways.^16^ IL-21 is primarily produced by activated CD4^+^ T cells and has pleiotropic effects.^17^^,^^18^^,^^19^ A growth-promoting effect of IL-21 has been observed in chronic lymphocytic leukemia,^20^^,^^21^ follicular lymphoma,^22^ Hodgkin’s lymphoma,^23^ and multiple myeloma^24^ and antiproliferative and proapoptotic effects on diffuse large B cell lymphoma.^25^ However, if and how IL-21/IL-21R signaling pathway affects AML LSCs and whether this knowledge might be translated into clinical application is still unknown.

In this work, we have identified IL-21/IL-21R signaling pathway as an important regulator of cell fate in LSCs, but not HSCs. We found that IL-21 is a positive prognostic marker for overall survival (OS) and that higher serum IL-21 levels correlate with better survival and higher rate of complete remission in patients that undergo high-dose chemotherapy. Our findings therefore suggest that promoting IL-21/IL-21R signaling on LSCs may be an approach to decrease stemness and increase differentiation in AML.

## Results

### IL-21/IL-21R signaling reduces murine L-GMPs *in vivo*

**Figure 1:**
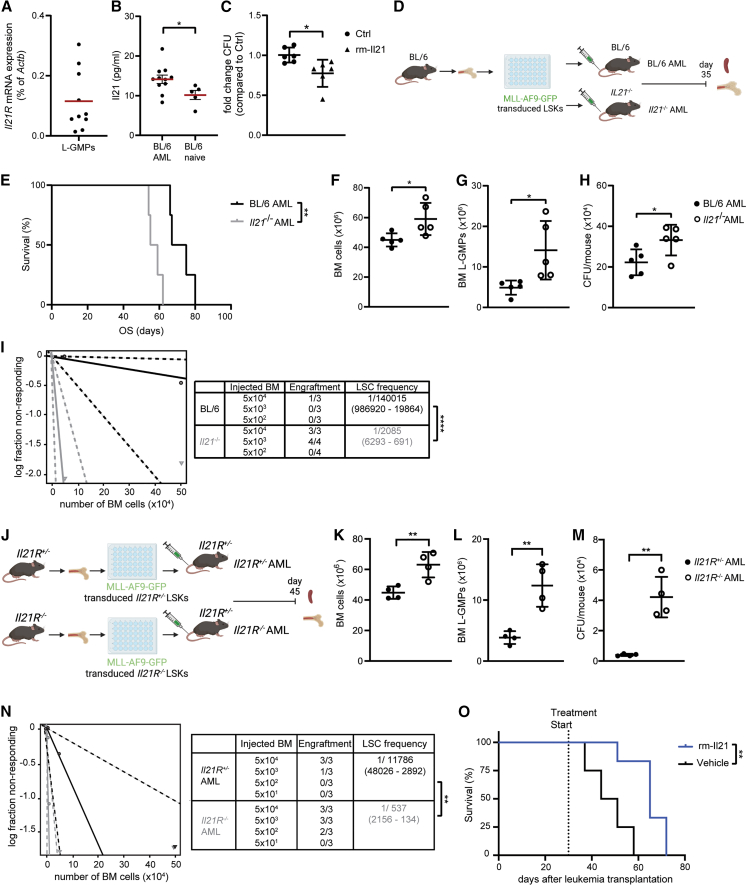
IL-21/IL-21R signaling reduces murine L-GMPs in vivo (A) *Il21R* mRNA expression (qRT-PCR) in FACS-sorted L-GMPs from the BM of BL/6 AML mice 35 days after leukemia transplantation (*n* = 10 mice). Red bar indicates the mean. Each dot represents the mean of two technical replicates. (B) IL-21 levels in BM samples from AML mice (*n* = 11 mice) and naive controls (*n* = 5 mice). Each dot represents the mean of two technical replicates. Data are shown as mean ± SEM. Statistics: Student’s t test. (C) Fold change colony-forming units from FACS-sorted L-GMPs cultured in methylcellulose for 7 days in the presence or absence of 300 pg/mL rm-IL-21 (*n* = 6 mice/group). Each dot represents the mean of two technical replicates. Pooled data from two independent experiments are shown and displayed as mean ± SD. Statistics: Student’s t test. (D) Experimental setup: 5 × 10^4^ MLL-AF9-GFP-transduced LSKs from the BM of BL/6 donors were injected intravenously into non-irradiated BL/6 and *Il21*^*−/−*^ recipients (BL/6 AML and *Il21*^*−/−*^ AML, respectively). Mice were sacrificed 35 days after leukemia transplantation and BM and spleen were analyzed. (E) MLL-AF9-GFP AML was induced in BL/6 and *Il21*^*−/−*^ recipients (*n* = 4 mice/group) and survival was monitored. Statistics: log rank test. (F and G) BM cellularity (F) and number of L-GMPs (G) in BM of BL/6 and *Il21*^*−/−*^ AML mice (*n* = 5 mice/group). Data are displayed as mean ± SD. Statistics: Student’s t test. (H) Colony-forming units per mouse. 5 × 10^4^ BM cells were plated into methylcellulose, and GFP^+^ colonies were enumerated 7 days later by inverted fluorescence microscopy (*n* = 5 mice/group). Data are displayed as mean ± SD. Statistics: Student’s t test. (F–H) One representative of four independent experiments is shown. (I) Extreme limiting dilution analysis. BM cells from BL/6 and *Il21*^*−/−*^ AML mice were injected at limiting dilutions into lethally irradiated (2 × 6.5 Gy) BL/6 recipients, and engraftment was assessed 30 days later. Statistics: χ2 test. (J) Experimental setup: 5 × 10^4^ MLL-AF9-GFP-transduced LSKs from the BM of *Il21R*^*+/−*^ and *Il21R*^*−/−*^ donors were injected intravenously into non-irradiated *Il21R*^*+/−*^ recipients (*Il21R*^*+/−*^ and *Il21R*^*−/−*^ AML, respectively). Mice were sacrificed 45 days after leukemia transplantation and BM and spleen were analyzed. (K–M) BM cellularity (K), number of L-GMPs in BM (L), and colony-forming units per mouse (M) (*n* = 4 mice/group). Data are displayed as mean ± SD. Statistics: Student’s t test. One representative of two independent experiments is shown. (N) Extreme limiting dilution analysis. BM cells from *Il21R*^*+/−*^ and *Il21R*^*−/−*^ AML mice were injected at limiting dilutions into lethally irradiated (2 × 6.5 Gy) BL/6 recipients and engraftment was assessed 30 days later. Statistics: χ2 test. (O) MLL-AF9-GFP AML was induced in BL/6 recipients (*n* = 4 mice/group). Thirty days after leukemia transplantation, mice were randomized to treatment with rm-IL-21 or vehicle and survival was monitored. Statistics: log rank test. One representative of four independent experiments is shown. ∗*p* < 0.05; ∗∗*p* < 0.01; ∗∗∗∗*p* < 0.0001. Abbreviations: L-GMPs, leukemic granulocyte-macrophage progenitors; LSKs, Lin^−^ Sca-1^+^ c-kit^+^; Lin, lineage; OS, overall survival; BM, bone marrow; CFU, colony-forming units; LSC, leukemic stem cell. See also Figures S1 and S2.

We analyzed the expression of the IL-21R on leukemic granulocyte-monocyte progenitors (L-GMPs), which represent the LSC population in mixed lineage leukemia (MLL)-AF9 (KMT2A-MLLT3) AML mice, and the level of IL-21 in the BM in a murine AML model.^26^ L-GMPs expressed the IL-21R, and IL-21 levels were increased in the BM of AML compared to naive mice (Figures 1A and 1B). IL-21 treatment reduced colony-forming capacity of L-GMPs, suggesting that L-GMPs can directly respond to IL-21 (Figure 1C). Colony formation reduction induced by IL-21 treatment on L-GMPs was dose dependent (Figure S1A).

Next, we transplanted MLL-AF9-GFP-transduced BL/6 lineage^−^Sca-1^+^c-kit^+^ cells (LSKs) into non-irradiated BL/6 mice and *Il21*^−/−^ mice (BL/6 AML and *Il21*^−/−^ AML, respectively) (Figure 1D). AML development was faster and resulted in significantly shorter survival for *Il21*^−/−^ compared to BL/6 AML mice (Figures S1B and 1E). To determine the role of IL-21/IL-21R signaling on L-GMPs, BL/6 and *Il21*^−/−^ AML mice were sacrificed 35 days after AML induction. Leukemia burden, as indicated by BM cellularity, numbers of MLL-AF9-GFP^+^Gr1^+^Cd11b^+^ leukemic cells in the spleen and BM, and leukemic blast frequency in the BM, was smaller in BL/6 AML mice compared to *Il21*^−/−^ AML mice (Figures 1F and S1C–S1F). In addition, the frequency of primitive AML cells (MLL-AF9-GFP^+^lin^−^ cells) was substantially reduced in BL/6 AML mice (Figure S1G). Similarly, the number of L-GMPs was significantly diminished in BL/6 AML mice phenotypically and functionally, as assessed by flow cytometry and colony formation assays (Figures 1G and 1H).

To functionally investigate leukemia-initiating cells *in vivo*,^27^ we transferred BM cells from primary BL/6 and *Il21*^−/−^ AML mice at titrated numbers into lethally irradiated secondary recipients. Extreme limiting dilution analysis^28^ revealed that the presence of IL-21 substantially reduced the frequency of L-GMPs in limiting dilution experiments *in vivo* by a factor of 67 (Figures 1I and S1H).

Comparable results on L-GMPs and AML development have been obtained when MLL-AF9-GFP-transduced and MLL-ENL-YFP-transduced *Il21R*^+/−^ and *Il21R*^−/−^ LSKs were transplanted into *Il21R*^+/−^ control mice (*Il21R*^+/−^ AML and *Il21R*^−/−^ AML, respectively) (Figures 1J–1N, S1I–S1L, and S2A–S2E), indicating that IL-21/IL-21R signaling on AML cells regulates leukemogenesis.

Importantly, *Il21R* deficiency on LSKs did not affect their repopulating capacity in steady-state and stress-induced hematopoiesis (Figures S2F and S2G).

These data suggest that *Il21/Il21R* signaling affects stem cell function in AML but not in normal and demand-adapted hematopoiesis.

### IL-21 treatment reduces disease development in MLL-AF9 AML mice

To demonstrate a role for IL-21 in the treatment of AML, we treated MLL-AF9 AML mice with overt leukemia daily with 20 μg recombinant mouse (rm)-IL-21 or vehicle in a 5 days on and 2 days off treatment schedule and assessed survival. 20 μg rm-IL-21 has previously been shown to affect tumor growth in syngeneic cancer models.^29^ rm-IL-21 treatment significantly prolonged the survival of MLL-AF9 mice (Figure 1O).

### IL-21/IL-21R signaling reduces stem cell maintenance and triggers differentiation-promoting signaling pathways in L-GMPs

**Figure 2:**
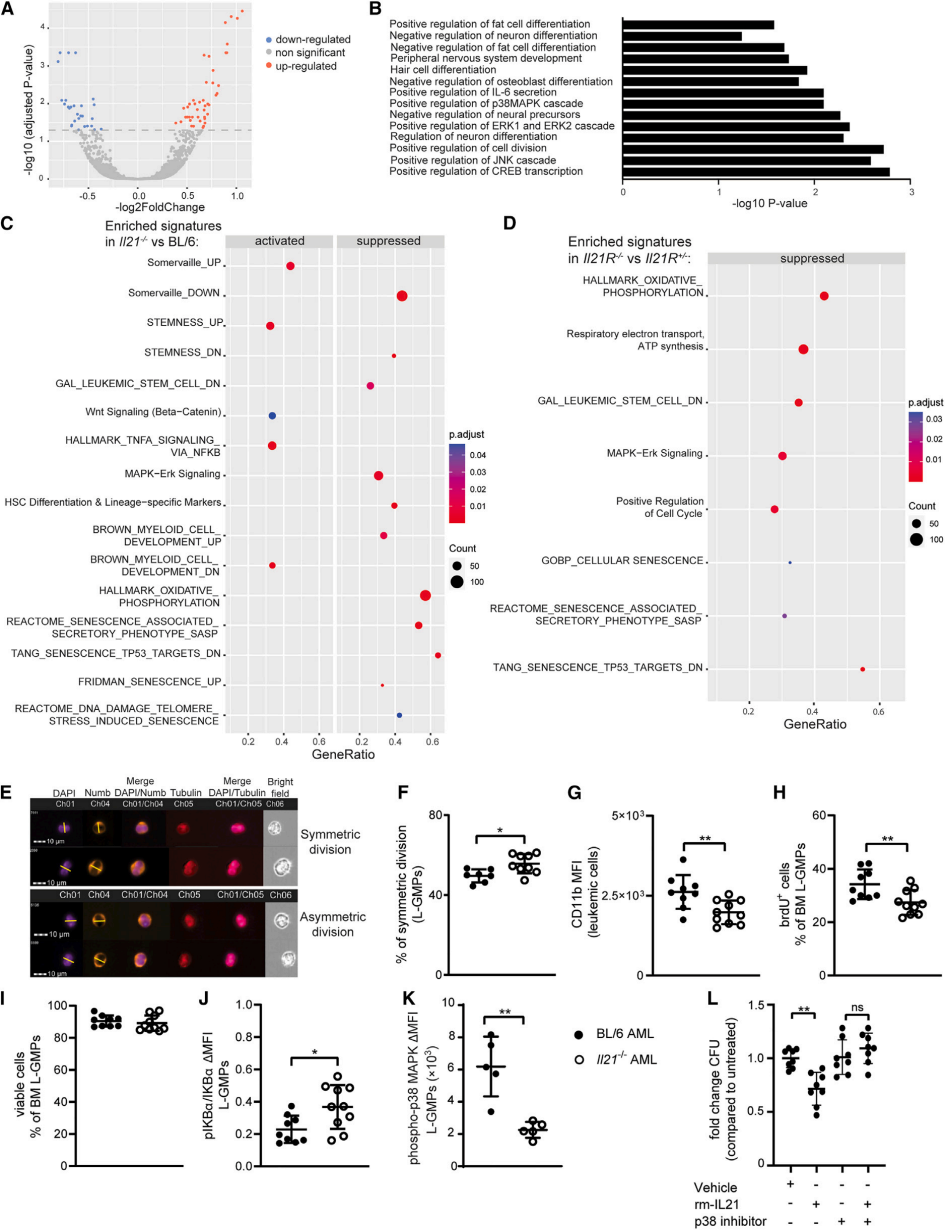
IL-21/IL-21R signaling reduces stem cell-related signaling pathways and triggers differentiation-promoting signaling pathways in L-GMPs (A) Volcano plot of differentially expressed genes in L-GMPs from BM of BL/6 and *Il21*^*−/−*^ AML mice (*n* = 3 mice/group). Log2 fold differences of gene expression levels in L-GMPs from BM of *Il21*^*−/−*^ AML mice versus L-GMPs from BM of BL/6 AML mice are shown. (B) Bar plot for the −log10 of the *p* value of selected GO terms (biological process), showing enriched pathways of differentially expressed genes. (C and D) Gene set enrichment analysis (GSEA) showed the activated and suppressed pathways in (C) L-GMPs from *Il21*^*−/−*^ AML mice versus L-GMPs from BL/6 AML mice and in (D) L-GMPs from *Il21R*^*−/−*^ AML mice versus L-GMPs from *Il21R*^*+/−*^ AML mice (*n* = 3 mice/group). A dot plot was generated to show the most significant enriched terms, with dot size indicating gene counts and dot color indicating the enrichment scores as adjusted *p* values. (E) Representative picture of Numb distribution in dividing FACS-purified L-GMPs from BM of BL/6 and *Il21*^*−/−*^ AML mice. Cells were analyzed by ImageStreamxMkII. Nuclei are stained with DAPI (in violet), α-tubulin is stained in red, and Numb in orange. Cell division plane (yellow line) was assigned based on α-tubulin and the cleavage furrow. (F) Quantification of L-GMPs from BM of BL/6 (*n* = 7) and *Il21*^*−/−*^ (*n* = 9) AML mice in symmetric cell division. Statistics: Student’s t test. (E and F) Two pooled independent experiments are shown (*n* = 3–5 mice/group). (G) CD11b mean fluorescence intensity (MFI) of MLL-AF9-GFP^+^ leukemic cells from BM of BL/6 and *Il21*^*−/−*^ AML mice (*n* = 9–10 mice/group). (H–J) Quantification of proliferating L-GMPs measured as brdU incorporation *in vivo* (H), cell viability measured as percentage of AnnexinV^−^ L-GMPs (I), NF-κB pathway activation (J) measured as ratio between protein expression of IκBα and its phosphorylated form pIκBα in L-GMPs from BM of BL/6 and *Il21*^*−/−*^ AML mice (*n* = 9–10 mice/group). (G–J) Two pooled independent experiments are shown (*n* = 5–6 mice/group). (K) Delta of the geometric MFI of phospo-p38 MAPK staining versus its isotype control on L-GMPs from BM of BL/6 and *Il21*^*−/−*^ AML mice (*n* = 5 mice/group). (L) FACS-purified L-GMPs from BL/6 AML mice were pre-treated with 10 nm/mL of the p38-MAPK inhibitor SB203580 or vehicle prior to overnight culture in the presence or absence of 300 pg/mL rm and plated in methylcellulose. Fold-change colony formation is depicted in the graph. Each dot represents the mean of two technical replicates. Pooled data from two independent experiments are shown and displayed as mean ± SD. Statistics: Student’s t test. ∗*p* < 0.05; ∗∗*p* < 0.01. Abbreviations: PC, principal component; L-GMPs, leukemic granulocyte-macrophage progenitors; MFI, mean fluorescence intensity; brdU, bromodeoxyuridine. See also Figure S3.

To analyze the molecular mechanism of how IL-21 affects stemness of L-GMPs, we performed bulk RNA sequencing (RNA-seq) analysis on L-GMPs derived from the BM of BL/6 AML and *Il21*^−/−^ AML mice. 72 genes were differentially expressed between BL/6 and *Il21*^−/−^ L-GMPs, with 44 and 28 genes being up- and down-regulated, respectively (Figure 2A). Gene ontology (GO) and gene set enrichment analysis (GSEA) revealed that IL-21/IL-21R signaling in L-GMPs reduced gene expression signatures related to proliferation, mitochondrial activity, and stemness, as well as stem cell-related signaling pathways such as WNT and nuclear factor κB (NF-κB), and promoted differentiation signatures (Figures 2B and 2C). In contrast, reactive oxygen species (ROS) production, MAPK signaling, and senescence signatures were activated by IL-21 signaling in L-GMPs (Figures 2B and 2C).

To identify the shared molecular mechanisms between L-GMPs derived from an IL-21-deficient microenvironment and L-GMPs lacking the IL-21R, we next performed bulk RNA-seq analysis on L-GMPs derived from *Il21R*^+/−^ AML and *Il21R*^−/−^ AML mice. Like L-GMPs from *Il21*^−/−^ AML mice, *Il21R*^*−/−*^ L-GMPs showed alterations in gene expression signatures related to stemness, ROS production, mitochondrial activity, senescence, and proliferation (Figure 2D).

### IL-21/IL-21R signaling reduces stemness of L-GMPs by favoring asymmetric division and activating the p38-MAPK signaling pathway

To confirm the RNA-seq results, we first addressed asymmetric division and symmetric renewal of L-GMPs and CD11b expression of bulk leukemia cells. We found that L-GMPs in the BM of *Il21*^−/−^ AML mice show higher symmetric division rate compared to BL/6 AML controls (Figures 2E and 2F), suggesting that *Il21/Il21R* signaling regulates the differentiation of L-GMPs through promotion of asymmetric cell division over symmetric renewal. The proportion of L-GMPs undergoing symmetric division was also reduced when L-GMPs were cultured *in vitro* in the presence of rm-IL-21, further confirming the direct effect of IL-21 promoting asymmetric cell division of L-GMPs (Figure S3A). In line with these findings, bulk leukemia cells in the BM of *Il21*^−/−^ AML mice had a lower expression of the differentiation marker CD11b compared to bulk leukemia cells in the BM of BL/6 AML mice (Figure 2G). In addition, L-GMPs in the BM of *Il21*^−/−^ AML mice diluted BrdU significantly more compared to controls in 48 h label-retaining experiments *in vivo*, which is indicative of a rapidly dividing L-GMP population. (Figure 2H). Furthermore, L-GMPs in the BM of *Il21*^−/−^ AML mice, in spite of identical cell viability, showed increased phosphorylation of IKBα and decreased phosphorylation of p38-MAPK, indicative of altered NF-κB and p38-MAPK signaling activity (Figures 2I–2K and S3B).

AML stem cell properties can also be defined based on ROS levels and mitochondrial dynamics. Consistent with a less differentiated AML phenotype, staining of intracellular ROS with the cell-permeant dye CellRox revealed a smaller percentage of CellRox^+^ L-GMPs in the BM of *Il21*^−/−^ AML mice compared to BL/6 AML mice (Figure S3C). In addition, staining with the mitochondrial probes MitoTracker and TMRM revealed, respectively, that L-GMPs in the BM of *Il21*^−/−^ AML mice had increased mitochondrial mass, without displaying changes in their mitochondrial membrane potential, which is consistent with LSC dependency on oxidative phosphorylation (OXPHOS) for their metabolism (Figures S3D and S3E).

To verify that the cellular processes and signaling cascades identified in *Il21*^*−/−*^ AML mice are also modulated in *Il21R*^*−/−*^ AML mice, we assessed CD11b expression on bulk leukemia cells as well as phosphorylation states of IKBα and p38-MAPK in L-GMPs from *Il21R*^−/−^ and control *Il21R*^+/−^AML mice. In line with the findings obtained in *Il21*^*−/−*^ AML mice, *Il21R*^−/−^ AML mice had a lower expression of CD11b on bulk leukemia cells (Figures S3F). Furthermore, phosphorylation of p38-MAPK in *Il21R*^*−/−*^ L-GMPs was significantly reduced compared to controls, while phosphorylation of IKBα was unchanged between the groups (Figures S3G–S3I).

To functionally demonstrate that the IL-21-mediated regulation of L-GMPs is dependent on p38-MAPK signaling, we incubated L-GMPs with rm-IL-21 after pre-incubation with the potent and selective p38-MAPK inhibitor SB203580^30^ and assessed colony formation *in vitro*. Effect of IL-21 on colony formation could be reverted by the blockade of p38-MAPK signaling (Figure 2L). Similarly, rm-IL-21-induced p38-MAPK phosphorylation and ROS levels increase in L-GMPs, which could all be reverted almost to control levels in the presence of the p38-MAPK inhibitor (Figures S3J and S3K). Direct *in vitro* exposure of L-GMPs to rm-IL-21 did not affect NF-κB signaling as indicated by unchanged levels of IKBα phosphorylation state (Figure S3L). These findings suggest that *Il21/Il21R* signaling regulates the cell fate of L-GMPs in AML by inducing differentiation, accumulation of ROS, and activation of the p38-MAPK signaling pathway.

### CD4^+^ T cell-derived IL-21 reduces stemness of murine LSCs *in vivo*

**Figure 3:**
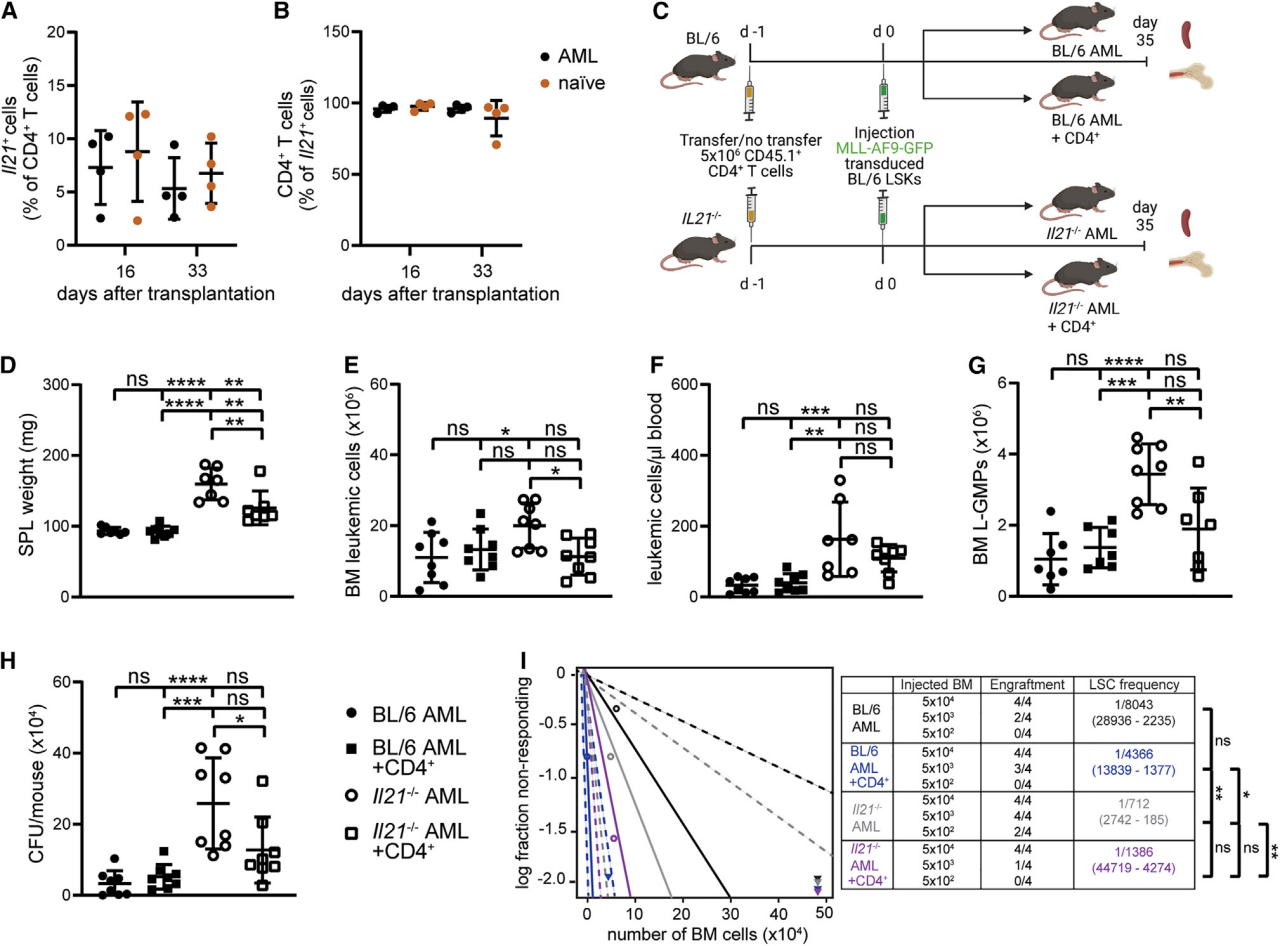
CD4^+^ T cell-derived IL-21 reduces murine LSCs *in vivo* (A and B) MLL-AF9 AML was induced in *Il21*^*mcherry*^ reporter mice. Frequencies of *mcherry-Il21*^+^ CD4^+^ T cells (A) and CD4^+^*mcherry-Il21*^+^ cells (B) were determined by flow cytometry 16 and 33 days after leukemia transplantation, in BM of AML and naive *Il21*^*mcherry*^ mice (*n* = 4 mice/group). (C) Experimental setup: 5 × 10^6^ CD4^+^ T cells were FACS-sorted from the spleens of CD45.1 mice and injected intravenously into two out of four experimental groups (non-irradiated BL/6 and *Il21*^*−/−*^ recipients) one day prior to leukemia transplantation. One day after, 5 × 10^4^ MLL-AF9-GFP-transduced LSKs from the BM of BL/6 donors were injected intravenously into all four experimental groups (BL/6 AML, *Il21*^−/−^ AML, BL/6 AML + CD4^+^, and *Il21*^−/−^ AML + CD4^+^, respectively). Mice were sacrificed 35 days after leukemia transplantation and BM and spleen were analyzed (*n* = 4 mice/group). (D–G) Spleen size (D), number of MLL-AF9-GFP^+^ leukemic cells in BM (E) and in peripheral blood (F), and number of L-GMPs (G) in the BM of BL/6 AML, *Il21*^−/−^ AML, BL/6 AML + CD4^+^, and *Il21*^−/−^ AML + CD4^+^ mice 35 days after leukemia transplantation. (H) Colony-forming units per mouse. 5 × 10^4^ BM cells were plated into methylcellulose and GFP^+^ colonies were enumerated 7 days later by inverted fluorescence microscopy. (D–H) Two pooled independent experiments are shown (*n* = 4 mice/group/experiment). Data are shown as mean ± SD. Statistics: one-way ANOVA followed by Tukey’s multiple comparisons test. (I) Extreme limiting dilution analysis. BM cells from BL/6 AML, *Il21*^−/−^ AML, BL/6 AML + CD4^+^, and *Il21*^−/−^ AML + CD4^+^ mice were injected at limiting dilutions into lethally irradiated (2 × 6.5 Gy) BL/6 recipients and engraftment was assessed 30 days later. Statistics: χ2 test. ∗*p* < 0.05; ∗∗*p* < 0.01; ∗∗∗*p* < 0.001; ∗∗∗∗*p* < 0.0001. Abbreviations: LSKs, Lin^−^ Sca-1^+^ c-kit^+^; SPL, spleen; L-GMPs, leukemic granulocyte-macrophage progenitors; CFU, colony-forming units. See also Figure S4.

To determine the source of IL-21 in AML, we induced MLL-AF9 AML in *Il21*^mcherry^ reporter mice.^31^ CD4^+^ T cells were identified as the primary source of IL-21 in BM, blood, and spleen of MLL-AF9 AML mice by flow cytometry (Figures 3A, 3B, and S4A). No difference in the frequency of IL-21-producing CD4^+^ T cells was observed between naive and AML mice. Similar results on IL-21 production by CD4^+^ T cells were obtained by quantitative reverse-transcription PCR (qRT-PCR) (Figure S4B). L-GMPs and CD45^−^lineage^-^ (CD45^−^lin^-^) BM cells, which comprise classical niche cells in AML such as MSCs and ECs, did not express IL-21 mRNA (Figures S4C and S4D).

To demonstrate that IL-21 derived from CD4^+^ T cells contributes to AML development, we adoptively transferred congenic CD45.1^+^ IL-21-proficient CD4^+^ T cells into BL/6 AML and *Il21*^−/−^ AML mice (Figure 3C). At the time of analysis, adoptively transferred CD4^+^ T cells could be detected in BM, spleen, and peripheral blood of BL/6 AML and *Il21*^−/−^ AML mice (Figures S4E–S4G). Transfer of CD4^+^ T cells into *Il21*^−/−^ AML significantly reduced leukemia burden and L-GMP numbers and frequency to levels comparable to BL/6 AML mice (Figures 3D–3I and S4H). These findings suggest that CD4^+^ T cell-derived IL-21 inhibits leukemia development and stemness in AML.

### IL-21 levels are elevated in the serum of patients at diagnosis and are a positive prognostic marker for OS

**Figure 4:**
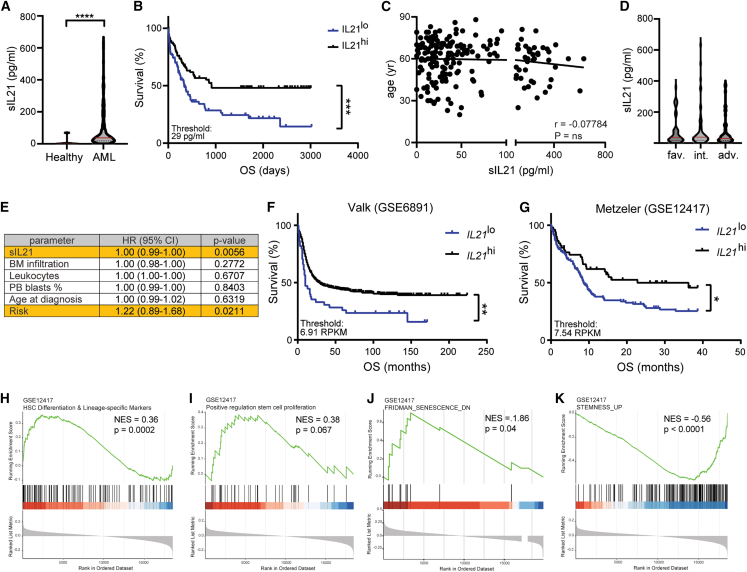
IL-21 is an independent positive prognostic marker for OS in AML (A) IL-21 levels in serum samples from newly diagnosed patients with AML (*n* = 193) and age-matched healthy controls (*n* = 10). Each dot represents the mean of two technical replicates. Red bars indicate the mean. Statistics: Mann-Whitney test. (B) Kaplan-Meier survival curves of the AML patients’ cohort (*n* = 193) divided into two groups at the IL-21 serum levels (sIL-21) threshold of 29 pg/mL. Statistics: log rank test. (C) Correlation of patients’ age with sIL-21 levels. Statistics: Pearson r test. (D) sIL-21 of patients in the different risk groups. Red bars indicate the mean. Statistics: one-way ANOVA. (E) Multivariate analysis for sIL-21 adjusted for BM infiltration, leukocyte counts, percentage of peripheral blood blasts, age, and risk group. Statistics: multiple Cox regression. (F and G) Two publicly available microarray datasets were analyzed for *IL21* mRNA expression levels and their association with prognosis. Statistics: log rank test. (F) Valk dataset, accession number GSE6891, sIL-21 threshold 6.91 RPKM. (G) Metzeler dataset, accession number GSE12417, sIL-21 threshold 7.54 RPKM. (H–K) Enrichment plots depicting significantly enriched gene sets in patients with high levels of IL-21. HSC differentiation and lineage-specific markers (H), positive regulation of stem cell proliferation (I), Fridman-senescence-DN (J), Stemness-UP (K). Gene sets were derived from the Metzeler dataset, accession number GSE12417. Normalized enrichment score (NES) and *p* value are indicated for each plot. ∗*p* < 0.05; ∗∗*p* < 0.01; ∗∗∗*p* < 0.001; ∗∗∗∗*p* < 0.0001. Abbreviations: OS, overall survival; yr, years; fav., favorable; int., intermediate; adv., adverse; HR, hazard ratio; CI, confidence interval; PB, peripheral blood; RPKM, reads per kilobase per million mapped reads; NES, normalized enrichment score. See also Figure S5.

We next determined IL-21 levels in the serum (sIL-21) of newly diagnosed patients with AML (Table S1). Because AML is primarily a disease of the elderly population, we initially verified in a publicly available resource^32^ that sIL-21 levels are not altered with age in healthy individuals (Figure S5A). In contrast, sIL-21 levels were significantly increased in 193 patients with AML compared to age-matched healthy controls (Figure 4A, mean sIL-21 81.6 and 7.9 pg/mL, respectively). Kaplan-Meier analysis revealed that patients with high levels of sIL-21 (≥29 pg/mL) survived substantially longer than patients with low levels of sIL-21 (Figure 4B). A similar effect of sIL-21 levels on OS was obtained when the patient cohort was subdivided in patients with low, intermediate, and high levels of sIL-21 (Figure S5B). Well-established risk factors for OS in AML such as patients’ age and cytogenetic/molecular risk group^5^ did not act as confounding factors in our analysis (Figures 4C and 4D) and sIL-21 could not be attributed to the differentiation state of AML based on the FAB classification (Figure S5C). Similarly, sIL-21 did not correlate with the numbers of CD4^+^ T cells in blood, which has been identified as a primary source of IL-21 (Figure S5D). Multivariate analysis for sIL-21 levels adjusted for patient age, risk group, blast percentage in blood and BM, as well as leukocyte counts, substantiated sIL-21 as an independent positive prognostic marker in AML (Figure 4E). Like sIL-21, high levels of *IL21* mRNA were associated with a favorable prognosis in two independent AML microarray datasets^33^^,^^34^ (Figures 4F and 4G). *IL21* mRNA did not correlate with *CD4* mRNA levels in either dataset (Figures S5E and S5F). However, GSEA analysis revealed that differentiation and proliferation signatures were increased and stemness and senescence signatures were decreased in patients with high levels of IL-21 (Figures 4H–4K).

These results identify sIL-21 and *IL21* mRNA as independent positive prognostic biomarkers for OS in AML.

### AML stem and progenitor cells but not normal hematopoietic stem and progenitor cells express the IL-21R

**Figure 5:**
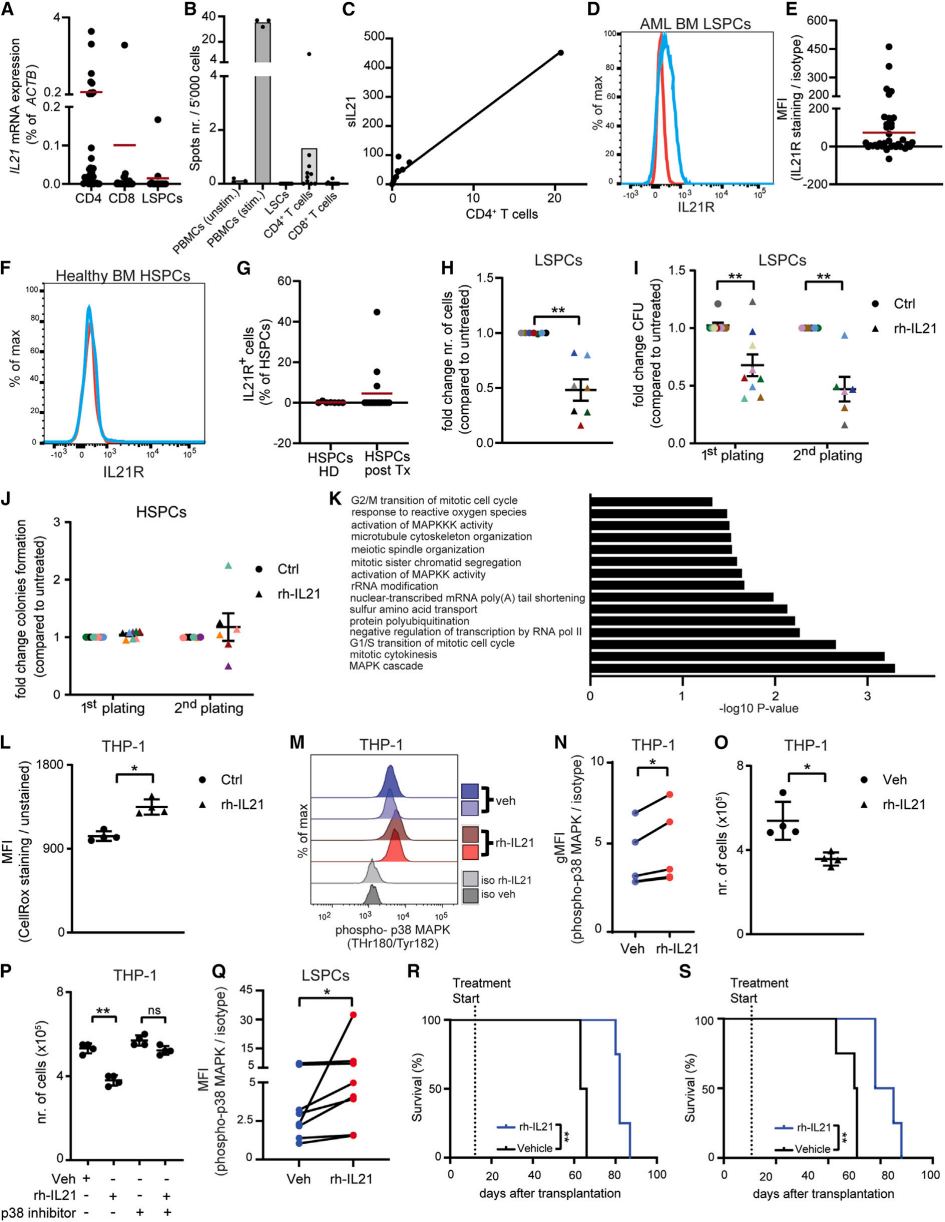
IL-21 is produced by CD4^+^ T cells and reduces cell growth and colony-forming capacity of AML stem and progenitor cells *in vitro* (A) *IL21* mRNA expression (qRT-PCR) in FACS-sorted CD4^+^ T cells (*n* = 32), CD8^+^ T cells (*n* = 27), and CD45^dim^SSC^lo^lin^−^CD34^+^ LSPCs (*n* = 13) from newly diagnosed patients with AML. Red bars indicate the mean. Statistics: one-way ANOVA. (B) IL-21 production by FACS-sorted matched CD4^+^ T cells, CD8^+^ T cells, and CD45^dim^SSC^lo^lin^−^CD34^+^ LSPCs from (*n* = 10) newly diagnosed patients with AML was measured by ELISPOT, using PHA-stimulated and unstimulated healthy donor-derived peripheral blood mononuclear cells (PBMCs) as an internal control. Spots count is shown from wells seeded with 5,000 cells. Each dot represents one technical replicates. (C) Linear correlation between the frequency of IL-21-producing CD4^+^ T cells detected by ELISPOT and the respective levels of sIL-21 measured by ELISA in these patients with AML. (D) Representative histogram of IL-21R (blue line) and relative isotype control (red line) staining on BM LSPCs. (E) Mean fluorescence intensity (MFI) quotient of IL-21R staining versus its isotype control on LSPCs (*n* = 35) from BM samples of newly diagnosed patients with AML. Red bar indicates the mean. (F) Representative histogram of IL-21R (blue line) and relative isotype control (red line) staining on BM HSPCs of healthy controls. (G) Percentage of HSPCs from the BM of healthy donors (*n* = 7) and patients with multiple myeloma who underwent allogeneic HSC transplantation (*n* = 15) that express IL-21R. Red bars indicate the mean. (H) Cell number (*n* = 7) of FACS-sorted LSPCs cultured *in vitro* for 72 h in the presence or absence of 100 pg/mL rh-IL-21. (I) LSPCs colonies were enumerated after two weeks of culture in methylcellulose in the presence or absence of 100 pg/mL rhIL-21 (two rounds of plating, *n* = 9). (J) HSPCs colonies were enumerated after two weeks of culture in methylcellulose in the presence or absence of 100 pg/mL rh-IL-21 (1^st^ plating, *n* = 8; 2^nd^ plating, *n* = 6). (H–J) Each dot represents the mean of three technical replicates. Different colors indicate different patients. Statistics: Student’s t test. Data are shown as mean ± SEM. (K) FACS-sorted CD45^dim^SSC^lo^lin^−^CD34^+^ LSPCs from three patients with AML were cultured *in vitro* in the presence or absence of 100 pg/mL rh-IL-21. After 72 h of culture, RNA was extracted and sequenced. The bar plot for the −log10 of the *p* value of selected GO terms shows enriched pathways of differentially expressed genes. (L–O) Intracellular reactive oxygen species measured by CellRox staining (L), histograms showing phosphorylation of p38 MAPK (phospho-p38 MAPK) (M), delta of the geometric MFI of phospo-p38 MAPK staining versus its isotype control (N), and number of THP-1 cells cultured *in vitro* for 72 h in the presence or absence of 1 nm/mL rh-IL-21 (O). Pooled data from four independent experiments are shown, with each dot representing the mean of three to five technical replicates. Statistics: paired Student’s t test. (P) THP-1 cells untreated or pretreated with 10 nm/mL of the p38-MAPK inhibitor SB203580 were cultured *in vitro* for 72 h in the presence or absence of 1 ng/mL rh-IL-21 and cell number was assessed. Pooled data from four independent experiments are shown and each dot represents the mean of three technical replicates. Data are shown as mean ± SD. Statistics: Student’s t test. (Q) Delta of the geometric MFI of phospo-p38 MAPK staining versus its isotype control of FACS-sorted CD45^dim^SSC^lo^lin^−^CD34^+^ LSPCs from seven patients with AML, cultured *in vitro* for 72 h in the presence or absence of 100 pg/mL rh-IL-21. T cells were used as an internal positive control. Each dot represents the mean of two technical replicates (see Figure S6G). (R and S) Patient-derived xenotransplants (AML 182 and AML 185, Table S1) were obtained by sublethally irradiating NSG-S mice and injecting 10^6^ FACS-purified primary CD45^dim^SSC^lo^ blasts from the BM of two newly diagnosed patients with AML via tail vein. Ten days after transplantation, mice were randomized to treatment regimen with rh-IL-21 or vehicle and survival was monitored. Statistics: log rank test. ∗*p* < 0.05; ∗∗*p* < 0.01; ∗∗∗∗*p* < 0.0001. Abbreviations: LSPCs, leukemic stem and progenitor cells; MFI, mean fluorescence intensity; HSPCs, hematopoietic stem and progenitor cells; HD, healthy donor; Tx, transplantation. See also Figure S6.

In human AML, *IL21* mRNA was mostly expressed by CD4^+^ T cells (22 out of 32 patients analyzed) but not CD8^+^ T cells and CD34^+^ AML stem and progenitor cells (LSPCs) in the BM of newly diagnosed patients with AML (Figures 5A and S6A for LSPCs gating strategy). ELISPOT analysis confirmed that primarily CD4^+^ T cells but not AML LSPCs and CD8^+^ T cells from the BM of patients with AML produce IL-21 (Figure 5B). The frequency of IL-21-producing CD4^+^ T cells correlated with IL-21 levels in the serum of newly diagnosed patients with AML (Figure 5C).

Next, we determined the expression of the IL-21R and its co-receptor CD132 on AML LSPCs. In human AML, the IL-21R was expressed on T cells but also on LSPCs in 21/35 BM samples and 12/30 blood samples by flow cytometry and qRT-PCR (Figures 5D, 5E, S6B, and S6C). CD132, the co-receptor for IL-21R, was expressed on LSPCs from all patients analyzed (data not shown). *IL21R* mRNA expression on LSPCs could not be associated with *IL21* mRNA expression by CD4^+^ T cells (Figure S6D). Normal hematopoietic stem and progenitor cells (HSPCs) derived from the BM of healthy donors did not express the IL-21R on the cell surface (Figures 5F, 5G, and S6E for HSPC gating strategy). Similarly, the vast majority of HSPCs (12/15) derived from the BM of these 15 patients with multiple myeloma 14 days after allogeneic stem cell transplantation did not express the IL-21R on the surface (Figure 5G).

### IL-21/IL-21R signaling on LSPCs inhibits cell growth and stemness *in vitro* through the activation of p38-MAPK signaling

Fluorescence-activated cell sorting (FACS)-purified CD45^dim^SSC^lo^lin^−^CD34^+^ LSPCs from different cytogenetic/molecular risk groups were cultured in the presence of 100 pg/mL of recombinant human (rh)-IL-21 for 72 h. 100 pg/mL of rh-IL-21 was selected because it resembles the mean concentration of IL-21 detected in the serum of patients with AML (Figure 4A). Addition of rh-IL-21 to the culture significantly reduced cell numbers per well without affecting cell viability (Figures 5H and S6F).

Similarly, rh-IL-21 treatment significantly reduced colony formation of LSPCs (Figure 5I). Replating revealed that this effect was maintained even in the absence of rh-IL-21 (Figure 5I). In addition, rh-IL-21 treatment resulted in increased colony size after replating (Figure S6G). rh-IL-21 treatment did not affect the clonogenic potential of HSPCs from healthy BM donors (Figure 5J).

To verify that p38-MAPK signaling is also active in primary human LPSCs, we incubated CD34^+^ LSPCs from 3 newly diagnosed patients with AML (Table S2) in the presence and absence of rh-IL-21 and performed bulk RNA-seq. GO analysis revealed that IL-21 triggered pathways related to cell cycle, ROS, and MAPK signaling (Figure 5K).

Culture of THP-1 AML cells, that express the IL-21R,^35^ in the presence of rh-IL-21 increased cellular ROS and p38-MAPK phosphorylation levels, resulting in reduced cell growth (Figures 5L–5O). Blockade of p38-MAPK signaling with the p38-MAPK inhibitor SB203580 restored the growth of THP-1 AML cells almost to the level of vehicle-treated THP-1 AML cells (Figure 5P). p38-MAPK phosphorylation levels increased in a similar fashion when primary LSPCs from patients with AML were incubated for 72 h with rh-IL-21 (Figures 5Q and S6H). These findings indicate that the IL-21/IL-21R interaction reduces cell growth and self-renewal of LSPCs, but not of normal HSPCs, through the accumulation of ROS and activation of p38-MAPK signaling.

To analyze the therapeutic potential of rh-IL-21 *in vivo*, we performed patient-derived xenograft (PDX) experiments.^36^ After 10 days of engraftment of human AML cells (Table S1, AML 182 and 185), NOD/SCID/γc^−/−^ mice expressing human IL-3, GM-CSF (CSF2), and SCF (KITLG) (NSG-S) were randomized to treatment with vehicle (Veh) or rh-IL-21 (20 μg, 5 days on and 2 days off). rh-IL-21 is not cross-reactive on mouse cells expressing the murine IL-21R.^37^ rh-IL-21 treatment significantly prolonged survival in xenotransplanted mice (Figures 5R and 5S).

### IL-21/IL-21R signaling promotes the sensitivity of AML LSCs to cytarabine treatment

**Figure 6:**
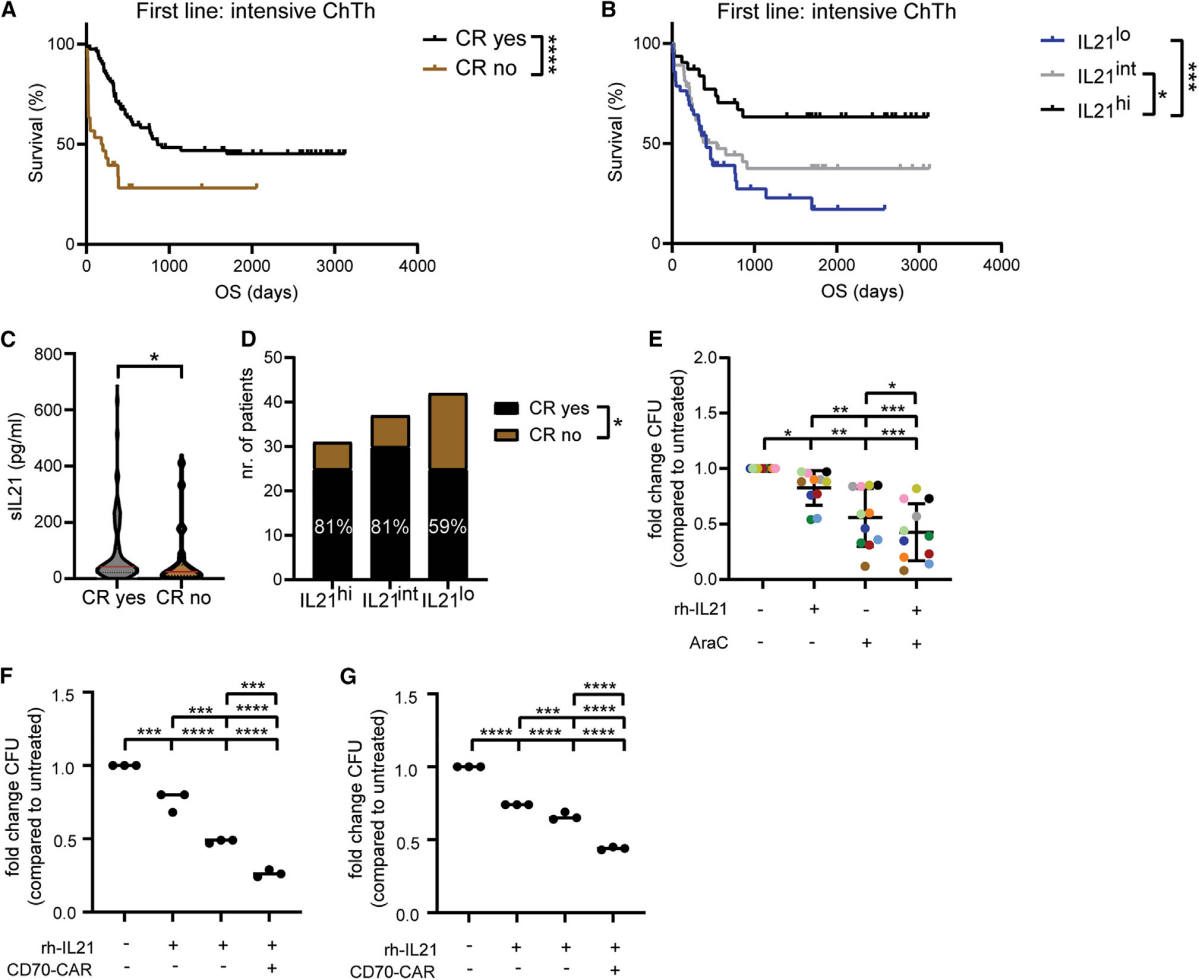
IL-21/IL-21R signaling promotes the sensitivity of AML LSCs to cytarabine treatment (A) Kaplan-Meier survival curves of patients with AML (*n* = 110) who received intensive chemotherapy (“7 + 3” regimen) as a first-line treatment divided into two groups based on complete remission (CR) achievement. Statistics: log rank test. (B) Kaplan-Meier survival curves of the AML patient cohort that received intensive chemotherapy (*n* = 110) divided into three groups at the sIL-21 threshold of 29 and 72 pg/mL. Statistics: log rank test. (C) sIL-21 levels in patients who achieved CR (*n* = 80) versus patients who did not achieve CR (*n* = 30). Red bars indicate the mean. Statistics: Mann-Whitney test. (D) CR rates in patients with high, intermediate, and low sIL-21 levels. Statistics: chi-square test. (E) LSPCs colonies were enumerated after two weeks of culture in methylcellulose in the presence or absence of 100 pg/mL rh-IL-21, 1 nM cytarabine (AraC), or combination. Each dot represents the mean of three technical replicates. Different colors indicate different patients (*n* = 11). Data are shown as mean ± SD. Statistics: one-way ANOVA followed by Tukey’s multiple comparisons test. (F and G) LSPCs colonies were enumerated after two weeks of culture in methylcellulose in the presence or absence of 100 pg/mL rh-IL-21, anti-CD70 CAR T cells (1:5 E:T ratio), or combination. Two patients are shown in each graph (each dot represents a technical replicate). Statistics: one-way ANOVA followed by Tukey’s multiple comparisons test. ∗*p* < 0.05; ∗∗*p* < 0.01; ∗∗∗*p* < 0.001; ∗∗∗∗*p* < 0.0001. Abbreviations: ChTh, chemotherapy; OS, overall survival; CR, complete remission; CFU, colony-forming units, AraC, cytosine arabinoside (or cytarabine). See also Figure S7.

Based on our findings, we hypothesized that IL-21/IL-21R signaling might render LSPCs more susceptible to chemotherapy. To test this hypothesis, we first analyzed sIL-21 levels only in 110 patients that received intensive chemotherapy (“7 + 3” regimen) as a first-line treatment. Importantly, patients that achieved complete remission (CR) after induction chemotherapy survived substantially longer compared to patients that did not achieve CR (Figure 6A). Kaplan-Meier analysis of this cohort further revealed that patients with high levels of sIL-21 (≥72 pg/mL) survived significantly longer than patients with low and intermediate levels of sIL-21 (Figure 6B). We found that sIL-21 levels at diagnosis are increased in patients that achieved CR compared to patients that did not achieve CR (Figure 6C). Patients’ age and cytogenetic/molecular risk category did not act as confounding factors in our analysis (Figures S7A and S7B). Furthermore, CR rate was found to be significantly higher in the subgroups with high and intermediate sIL-21 levels compared to the subgroup with low sIL-21 levels (81% versus 59%) (Figure 6D). In contrast, sIL-21 levels at diagnosis did not correlate with OS of patients that received first-line palliative treatment (Figure S7C).

Next, we incubated FACS-sorted LSPCs from different newly diagnosed patients with AML with cytarabine and rh-IL-21 for 24 h prior to culturing them in methylcellulose. We observed that the combination of IL-21 and cytarabine induced a stronger reduction in LSPC colony-forming capacity when compared with IL-21 or cytarabine single treatments (Figure 6E).

To determine whether IL-21 also sensitizes LPSCs to cell-based therapies currently used in hematological malignancies, we treated LPSCs from two newly diagnosed patients with AML with CAR T cells targeting the AML surface antigen CD70 in the presence and absence of rh-IL-21. CD70 has been identified as a valid target in the field of AML.^38^ Co-culture of CD70-targeting CAR T cells in the presence of rh-IL-21 significantly reduced the colony formation of LSPCs compared to monotherapy (Figures 6F and 6G).

These findings support the hypothesis that IL-21/IL-21R signaling contributes to a better response to chemotherapy and is associated with higher CR rates in patients with AML and support the notion that IL-21 could also be applied to other combination approaches in the field of AML.

## Discussion

Quiescent, therapy-resistant LSCs are the major cause of relapse after initially successful chemotherapy in AML.^39^^,^^40^^,^^41^ Novel methods effectively eradicating LSCs are an unmet medical need.

Self-renewal is a key feature of both normal and malignant stem cells which allows to maintain and expand the stem cell pool through, respectively, asymmetric division or symmetric renewal.^28^^,^^42^ Stem cell-related gene signatures are considered as poor prognostic markers for response to therapy and OS in AML.^43^^,^^44^^,^^45^ High frequencies and numbers of LSCs at diagnosis predict therapy resistance and negatively correlate with outcome.^46^^,^^47^ These stem cell-related signatures are frequently sustained at the expense of differentiation-promoting events, resulting in a block of differentiation and senescence of AML cells.^48^ All-trans retinoic acid is the first approved drug that induces the differentiation of blasts in a specific subtype of AML.^49^ In addition, inhibition of isocitrate dehydrogenase (IDH) 1 and 2 in patients with AML carrying mutations in IDH1 or 2 induces differentiation of AML cells.^50^ Recently, several other signaling cascades have been identified that may promote the differentiation of AML cells.^38^^,^^51^^,^^52^^,^^53^

We show that activation of IL-21/IL-21R signaling favors asymmetric cell division over symmetric renewal in LSCs. This change in division pattern resulted in a reduced pool of AML LSCs through promotion of differentiation, as indicated by increased expression of the differentiation marker CD11b and increased intracellular ROS content, as well as a reduction of LSC frequency in secondary transplantation experiments. This is in line with recent findings that suggested a correlation between metabolic state, cellular ROS, and the differentiation status of HSCs and LSCs. Several studies showed that LSCs have increased mitochondrial mass compared to their healthy counterparts and rely on OXPHOS for energy production.^53^^,^^54^^,^^55^ Nevertheless, through high activity of ROS-removing pathways, they can maintain very low ROS levels, a characteristic of self-renewing quiescent stem cells, whereas elevated ROS levels can push cells out of quiescence and promote differentiation.^54^^,^^55^^,^^56^^,^^57^^,^^58^^,^^59^

Many signaling pathways have been shown to promote stemness or differentiation of HSCs and LSCs.^60^^,^^61^^,^^62^ ROS modulate some of these molecules with reported function in stem cell maintenance and differentiation such as p38-MAPK. Activation of the p38-MAPK pathway by ROS is crucial in limiting the lifespan and functionality of HSCs.^61^^,^^62^ Treatment of HSCs with a p38-MAPK inhibitor improved the lifespan of HSCs.^61^ In complementary experiments, HSC-specific phosphorylation of p38-MAPK resulted in HSC activation and loss in self-renewal.^62^ ROS-low HSCs were shown to have higher self-renewal potential, whereas ROS-high HSCs exhausted faster in serial transplantation assays than their ROS-low counterparts due to increased activation of the p38-MAPK.^63^ In myelodysplastic syndromes (MDSs), the p38-MAPK pathway was identified as negative regulator of primary human MDS progenitor cell differentiation.^64^ Cytokines together with ROS have been identified as major inducers of p38-MAPK signaling-mediated differentiation in many different cell types.^65^ We show that the cytokine IL-21 promotes p38-MAPK signaling in AML LSCs resulting in reduced stemness and increased ROS levels in LSCs. The increased levels of ROS most likely must be considered an indicator of a more differentiated AML cell population with reduced stem cell potential rather than a direct consequence of active IL-21 signaling, as underscored by our main finding that IL-21 signaling reduces the amount of functional LSCs. IL-21 has been previously shown to induce related MAPK signaling pathways. For example, IL-21-induced ERK1/2 phosphorylation has previously been demonstrated in monocytes, THP-1 AML cells,^35^ and multiple myeloma cell lines.^24^ It is reasonable to hypothesize that in addition to p38, related kinases such as ERK1/2 and JNK may be activated by IL-21. However, functional experiments using the selective p38-MAPK inhibitor SB203580 completely rescued the IL-21-induced effect on L-GMPs and AML cell lines, suggesting that IL-21 primarily induces p38-MAPK signaling in LSCs.

AML is a highly complex and heterogeneous disease that arises from the stepwise acquisition of somatic mutations, including chromosomal aberrations and single-nucleotide variants.^5^ To develop a clinical application in AML, it would therefore be necessary to unravel which AML subtypes preferentially respond to IL-21 treatment. Our findings are mostly based on the functional experiments in MLL-driven AML mouse models, few PDX AML model, and a limited number of primary human AML samples with diverse cytogenetic and molecular aberrations. In addition, IL-21R expression could only be detected on 60% of AML samples by flow cytometry. Nevertheless, the effect on colony formation *in vitro* was observed consistently across samples, regardless of IL-21R expression levels. Although we did not demonstrate the absence of IL-21 effect on IL-21R-negative AML patient samples, we did show that healthy HSCs from the BM of healthy donors, which do not express IL-21R, do not respond to IL-21. Therefore, further investigations on the role of IL-21/IL-21R signaling on IL-21R-negative LSC and in specific AML subtypes are warranted.

IL-21 is secreted by multiple immune cell types and is variously involved in immune responses.^17^^,^^18^ Therefore, the induction of IL-21/IL-21R signaling on LSCs depends on IL-21 provided by cells of the immune microenvironment. In our study, analysis of IL-21 mRNA and protein levels of immune cells and AML cells combined with adoptive transfer experiments of CD4^+^ T cells identified CD4^+^ T cells as the primary source of IL-21 and CD4^+^ T cell-derived IL-21 as a negative regulator of stemness in AML. The mechanisms how IL-21 secretion and release are induced in patients with leukemia are currently unknown.

We found that IL-21 serum levels in patients with AML are increased compared to healthy controls and serve as an independent positive prognostic marker for OS. Serum IL-21 levels correlated with the frequency of IL-21-producing CD4^+^ T cells in the BM of newly diagnosed patients with AML and could therefore be used clinically as a surrogate biomarker to address the stemness signature of a patient’s AML blasts and to predict outcome.

Even though we could show IL-21 protein expression in murine and human CD4^+^ T cells in AML by different techniques and IL-21 in serum of patients with AML, IL-21 mRNA expression could not be detected in any of the cells in publicly available single-cell RNA-seq data from patients with AML and healthy donors.^66^ This discrepancy could be explained by the fact that single-cell RNA-seq is not sufficiently sensitive to allow for the analysis of mRNA from low-abundant transcripts such as cytokine genes.

Resistance to standard induction chemotherapy is one of the key features of LSCs and is accountable for relapse.^67^ One possibility to increase the chemotherapy sensitivity in AML could be the promotion of differentiation-promoting signaling cascades in LSCs. In this study, we document that high-dose chemotherapy was more effective in patients with high levels of sIL-21 at diagnosis as illustrated by a significantly higher rate of CR and prolonged OS.

In AML, chemoresistant AML cells have lower ROS levels in response to cytarabine.^68^ LSCs are protected from chemotherapy-induced cell death due to the activation of master regulators such as NRF2, which are involved in neutralizing cellular ROS and restoring redox balance.^69^ A recent study showed that standard induction chemotherapy leads to the elevation of ROS, but does not efficiently target LSCs, indicating that increased ROS levels alone are not sufficient to compromise LSCs’ viability or function.^55^ Because we found that IL-21/IL-21R signaling in AML LSCs increases ROS and promotes proliferation and asymmetric division, we hypothesized that IL-21 could render AML LSCs more susceptible for chemotherapy. Further investigations involving primary AML LSCs *in vitro* indeed demonstrated that activation of IL-21/IL-21R signaling renders LSCs more susceptible to cytarabine treatment. Another explanation for the increased sensitivity of LSCs to cytarabine after activation of IL-21 signaling may be the reversal of cytarabine-induced senescence. Cytarabine has recently been shown to induce a reversible senescent phenotype in AML LSCs.^70^ Our results indicate that IL-21/IL-21R signaling also activates senescence signatures in AML LSCs leading us to conclude that the increased sensitivity of LSCs in the combination regimen is not due to the reversal of senescence in AML LSCs by IL-21 signaling.

Attempts to evaluate the therapeutic potential of systemic delivery of rm-IL-21 to AML mice via adeno-associated viral vectors and to induce high levels of IL-21 failed due to IL-21-associated toxicities (data not shown). In patients with solid tumors, issues with hepatic or gastrointestinal toxicities have led to discontinuation of IL-21’s clinical development for systemic administration.^71^ In contrast, treatment with low-dose IL-21 significantly prolonged survival in AML PDX and MLL-AF9 mice without inducing toxicity. This is in line with previous findings, where low-dose IL-21 treatment was used to treat solid tumors in preclinical mouse models^29^ and identify low-dose IL21 as potential approach for the treatment of patients with cancer. Because many different cell types express the IL-21R, systemic IL-21 treatment may not only act on LSCs but most likely will also affect other IL-21R-expressing cell types such as CD8^+^ T cells and natural killer (NK) cells and may therefore improve the immune control of AML.

As an increasing number of selective drug delivery and targeted immunotherapy strategies are being currently developed and optimized, a potential exists for translating IL-21 into clinical application, for example by combination with standard-of-care treatment regimens as demonstrated in our study or by mean of bi- or tri-specific antibodies or chimeric antigen receptor (CAR) T cells that target AML LSCs. Indeed, we demonstrate in this study that combining IL-21 treatment with CAR T cells targeting CD70 may eliminate AML LSPCs more efficiently than CAR T cell monotherapy.

In summary, CD4^+^ T cell-derived IL-21 reduces stemness and therapy resistance of AML LSCs by inhibition of cytokine-induced p38-MAPK signaling and by promoting asymmetric cell division. Therefore, stimulating the IL-21/IL-21R signaling pathway may be an immunotherapeutic approach that allows the selective elimination of LSCs.

### Limitations of the study

This study has demonstrated a role for CD4^+^ T cell-derived IL-21 in the regulation of AML LSCs in mice and humans. These findings are primarily based on experiments in MLL-driven AML mouse models, a few PDX AML models, and a limited number of primary human AML samples with different cytogenetic and molecular aberrations. Furthermore, IL-21R expression could be only detected in 60% of AML samples, yet all samples tested in functional assays responded to IL-21 treatment *in vitro*. Therefore, further investigations in other experimental AML models, on IL-21R-negative AML samples and LSCs from specific AML subtypes, are required to better understand the potential of IL-21 treatment in AML. In addition, although this study provides evidence that low-dose IL-21 treatment prolongs the survival of AML mice in syngeneic and AML PDX models, systemic IL-21 treatment may not only act on LSCs but most likely also affect other IL-21R-expressing cell types such as CD8^+^ T cells and NK cells, and thus improve immune control of AML. Further studies are needed to dissect the contribution of anti-leukemic immunity to AML development after systemic IL-21 treatment.

## Resource availability

### Lead contact

Further information and requests for resources and reagents should be directed to and will be fulfilled by the lead contact, Carsten Riether (carsten.riether@insel.ch).

### Materials availability

All unique reagents generated in this study are available from the lead contact without restriction.

### Data and code availability

All RNA-seq data compiled for this study are made publicly available on the Gene Expression Omnibus (GEO) website (https://www.ncbi.nlm.nih.gov/geo/) under the accession number GSE241170, GSE241171, and GSE241172. This study does not include the development of new code. Any additional information required to re-analyze the data reported is available from the lead contact upon request.

## Acknowledgments

We thank the staff of the FACS lab (Department for BioMedical Research (DBMR), University of Bern, Switzerland) for providing excellent technical assistance. This work was supported by grants from the 10.13039/501100013362Swiss Cancer Research (KFS-4389-02-2018), 10.13039/501100001711Swiss National Science Foundation (310030_179394), and ETH Foundation (LC-01-22).

## Author contributions

Conceptualization, C.R.; methodology, V.R., I.K., M.K., M.-N.K., A.F.O., and C.R.; investigation, V.R., M.H., L. Taylor, L. Tortola, S.H., N.S., H.L., S.V., I.K., R.R., and U.B.; writing – original draft, V.R. and C.R.; writing – review, all authors; supervision, C.R.

## Declaration of interests

The authors declare no competing interests.

## STAR★Methods

### Key resources table

**Table:** 

REAGENT or RESOURCE	SOURCE	IDENTIFIER
**Antibodies**		

Anti-mouse Ly-6C/G (Gr1-1)-APC	BioLegend	Cat# 108412; RRID: AB_313377
Anti-mouse CD11b-PE-Cy7	BioLegend	Cat# 101216; RRID: AB_312798
Anti-mouse CD11b- PerCP-Cy5.5	BioLegend	Cat# 101228; RRID:AB_893232
Anti-mouse CD19-APC-Cy7	BioLegend	Cat# 115530; RRID:AB_830707
Anti-mouse Ly-6A/E (Sca-1)-PerCP-Cy5.5	BioLegend	Cat# 122524; RRID:AB_893617
Anti-mouse Ly-6A/E (Sca-1)-APC-Cy7	BioLegend	Cat# 108126; RRID: AB_10645327
Anti-mouse CD117 (c-kit)-APC-Cy7	BioLegend	Cat# 105826; RRID:AB_1626278
Anti-mouse CD16/32 (Fcγ)-PE-Cy7	BioLegend	Cat# 101318; RRID:AB_2104156
Anti-mouse CD4-BV650	BioLegend	Cat# 100555; RRID:AB_2562529
Anti-mouse CD8a-Alexa Fluor® 700	BioLegend	Cat# 100730; RRID:AB_493703
Anti-mouse CD19-biotin	BioLegend	Cat# 115503; RRID: AB_313638
Anti-mouse CD11b-PE-Cy7	BioLegend	Cat# 101216; RRID: AB_312799
Anti-mouse CD3ε-biotin	BioLegend	Cat# 100304; RRID: AB_312669
Anti-mouse Ter119-biotin	BioLegend	Cat# 116203; RRID: AB_313704
Anti-mouse CD34-eFluor® 450	ThermoFisher Scientific	Cat# 48-0341-82; RRID: AB_2043837
Anti-mouse CD117 (c-kit)-BUV395	BD Biosciences	Cat# 564011; RRID:AB_2738541
Anti-human CD34-APC	BioLegend	Cat# 343608; RRID: AB_2228972
Anti-human CD38-PE-Cy7	BioLegend	Cat# 303515; RRID:AB_1279235
Anti-human CD2-biotin	Biolegend	Cat# 300204; RRID: AB_314028
Anti-human CD14-biotin	BioLegend	Cat# 325624; RRID: AB_2074052
Anti-human CD16-biotin	BioLegend	Cat# 302004; RRID: AB_314204
Anti-human CD19-biotin	BioLegend	Cat# 302203; RRID: AB_314233
Anti-human CD56-biotin	BioLegend	Cat# 318320; RRID: AB_893390
Anti-human CD235ab-biotin	BioLegend	Cat# 306618; RRID: AB_2565773
Anti-human CD4-APC-Cy7	BioLegend	Cat# 317417; RRID: AB_571946
Anti-human CD8-PerCP-Cy5.5	BioLegend	Cat# 344710; RRID: AB_2044010
Anti-human CD45-V500-C	BD Biosciences	Cat# 647449; RRID: AB_2870319
Anti-human CD360 (IL21R)	Miltenyi	Cat# 130-101-477; RRID: AB_2657745
Human IgG1, REA Control Antibody (S)	Miltenyi	Cat# 130-113-438; RRID: AB_2733893
Rabbit anti-IκBα (44D4) mAb (unconjugated)	Cell Signaling Technology	Cat# 4812; RRID: AB_10694416
Rabbit anti-pIκBα (14D4) mAb (unconjugated)	Cell Signaling Technology	Cat# 2859; RRID: AB_561111
Anti-rabbit IgG (H + L), F(ab')2 Fragment-Alexa Fluor® 647	Cell Signaling Technology	Cat# 4414; RRID: AB_10693544
Goat anti-Numb polyclonal Ab (unconjugated)	Abcam	Cat# ab4147; RRID: AB_304320
Mouse anti-Tubulin mAb (unconjugated)	Abcam	Cat# ab7291; RRID: AB_2241126
AlexaFluor®568 donkey-anti goat IgG H&L	Abcam	Cat# ab175704; RRID: AB_2725786
AlexaFluor®647 rabbit-anti mouse IgG H&L	Abcam	Cat# ab150127
PE anti-p38 MAPK Phospho (Thr180/Tyr182)	BioLegend	Cat# 690203; RRID: AB_2832849
PE Mouse IgG1, κ Isotype Ctrl Antibody	BioLegend	Cat# 400139; RRID: AB_493443

**Biological samples**		

BM aspirates from untreated, newly diagnosed AML patients	Department of Hematology and Central Hematology Laboratory, Inselspital, Bern University Hospital and University of Bern	N/A
PB from untreated, newly diagnosed AML patients	Department of Hematology and Central Hematology Laboratory, Inselspital, Bern University Hospital and University of Bern	N/A
BM from healthy donors (orthopedic patients who underwent Vertebroplasty)	Inselspital, Bern University Hospital and University of Bern	N/A

**Chemicals, peptides, and recombinant proteins**		

MethoCult™ H4435 Enriched for human cells	STEMCELL Technologies	Cat# 04435
MethoCult™ M3134 for mouse cells	STEMCELL Technologies	Cat# 03134
Human Methylcellulose Serum-Free Enriched Media	R&D Systems	Cat# HSC005SF
Mouse recombinant IL-3	Miltenyi	Cat# 130-099-508
Mouse recombinant IL-6	Miltenyi	Cat# 130-096-682
Mouse recombinant SCF	Miltenyi	Cat# 130-101-697
Mouse recombinant Flt3l	Miltenyi	Cat# 130-094-038
Human recombinant IL-7	Miltenyi	Cat# 130-095-367
Human recombinant IL-15	Miltenyi	Cat# 130-095-760
T cell TransAct™	Miltenyi	Cat# 130-128-758
TexMACS™ Medium	Miltenyi	Cat# 130-097-196
Human Insulin Actrapid	Novo Nordisk	Cat# 8-0201-83-201-3
Human holo-transferrin	Prospec	Cat# PRO-315
StemSpan™ Medium	STEMCELL Technologies	Cat# 09605
StemSpan™ Cytokine Cocktail (CC)-100	STEMCELL Technologies	Cat# 02690
APC Streptavidin	BioLegend	Cat# 405207
FITC Streptavidin	BioLegend	Cat# 405202
Pacific Blue™ Annexin V	BioLegend	Cat# 640918
V500 Streptavidin	BD Biosciences	Cat# 561419, RRID: AB_10611863
Fixable Viability Dye eFluor® 506	ThermoFisher Scientific	Cat# 65-0866-14
Fixable Viability Dye eFluor® 450	ThermoFisher Scientific	Cat# 65-0863-14
Human recombinant IL-21	Sigma-Aldrich	Cat# SRP3087
Cytosine β-D-arabinofuranoside	Sigma-Aldrich	Cat# C1768
DAPI	Merck	Cat# 10236276001
Dako Wash	Agilent Technologies	Cat# S300685-2
Dako Antibody Diluent	Agilent Technologies	Cat# S080983-2
CellROX™ Deep Red Reagent	ThermoFisher Scientific	Cat# C10422
Tetramethylrhodamine, Methyl Ester, Perchlorate (TMRM™)	ThermoFisher Scientific	Cat# T668
MitoTracker™ Red FM	ThermoFisher Scientific	Cat# M22425
SB203580 p38 MAPK inhibitor	STEMCELL Technologies	Cat# 72222
True-Phos™ Perm Buffer	BioLegend	Cat# 425401
PEG-it™ Virus Precipitation Solution	VWR	Cat# MSPP-LV810A1

**Critical commercial assays**		

APC BrdU Flow Kit	BD Biosciences	Cat# 552598; RRID: AB_2861367
RNA Easy Micro Kit	Qiagen	Cat# 74004
Quick-RNA Microprep Kit	Zymo Research	Cat# R1051
ELISA MAX™ Deluxe Set Human IL-21	BioLegend	Cat# 433804
ELISPOT Plus: Human IL-21	Mabtech	Cat# 3540-4APW-2

**Deposited data**		

RNA-seq data		GEO: GSE241170, GSE241171, GSE241172

**Experimental models: Cell lines**		

THP-1	ATCC	TIB-202™

**Experimental models: Organisms/strains**		

Mouse: C57BL/6J	Charles River	MGI: 3028467
*Il21R*^−/−^ (B6.129-Il21rtm1Kopf/J)		MGI: 5435248
*Il21*^−/−^ (B6.129S-Il21tm1Lex/Mmucd)		MGI: 4843320
*Il21*^*mCherry*^(B6.Cg-Tg(Il21-mCherry)1Wjl/Mmnc)		MGI: 5478531

**Recombinant DNA**		

Plasmid pMDLg/pRRE		Addgene #12259
Plasmid pRSV-Rev		Addgene #12253
Plasmid pCMV-VSV-g		Addgene #8454
CD19-CAR plasmid		Addgene #200671

**Software and algorithms**		

FlowJo™ software v.10.6	TreeStar	N/A
GraphPad Prism® software v9.0	GraphPad	N/A
ELDA Software	Hu et al.^21^	http://bioinf.wehi.edu.au/software/elda/
fastqc v. 0.11.9		https://www.bioinformatics.babraham.ac.uk/projects/fastqc/
RSeQC v. 4.0.0	Wang et al.^72^	N/A
HiSat2 v. 2.2.1	Kim et al.^73^	N/A
FeatureCounts v. 2.0.1	Liao et al.^74^	N/A
DESeq2 v.1.32.0	Love et al.^75^	N/A
TopGo v. 2.44.0		https://bioconductor.org/packages/release/bioc/html/topGO.html
ClusterProfiler v. 4.0.2	Yu et al.^76^	N/A
R 4.1.0		https://www.R-project.org/
INSPIRE® software		https://www.merckmillipore.com/
IDEAS® software		https://www.merckmillipore.com/
Kaluza analysis software	Beckman Coulter Life Sciences	N/A

**Other**		

HALLMARK OXIDATIVE PHOSPHORYLATION		gsea-msigdb.org
Somervaille_UP	Somervaille et al.^26^	N/A
Somervaille_DOWN	Somervaille et al.^26^	N/A
STEMNESS_UP		gsea-msigdb.org
STEMNESS_ DOWN		gsea-msigdb.org
GAL_LEUKEMIC STEM CELL_DOWN		gsea-msigdb.org
BROWN_MYELOID_CELL_DEVELOPMENT_DN		gsea-msigdb.org
BROWN_MYELOID_CELL_DEVELOPMENT_UP		gsea-msigdb.org
HALLMARK_TNFA_SIGNALING_VIA_NFKB		gsea-msigdb.org
HSC Differentiation and Lineage-specific markers		pathcards.genecards.org
Wnt Signaling (beta-Catenin)		pathcards.genecards.org
MAPK-Erk Signaling		pathcards.genecards.org
POSITIVE REGULATION OF CELL CYCLE		gsea-msigdb.org
Respiratory electron transport_ATP synthesis		pathcards.genecards.org
FRIDMAN_SENESCENCE_UP		gsea-msigdb.org
TANG_SENESCENCE_TP53_TARGETS_DN		gsea-msigdb.org
REACTOME_SENESCENCE_ASSOCIATED_SECRETORY_PHENOTYPE_SASP		gsea-msigdb.org
REACTOME_DNA_DAMAGE_TELOMERE_STRESS_INDUCED_SENESCENCE		gsea-msigdb.org
GOBP_CELLULAR_SENESCENCE		gsea-msigdb.org

### Experimental model and study participant details

#### Mice

C57BL/6J (BL/6) mice and NOD SCID gamma (NSG-S) mice were purchased from Charles River Laboratories (Sulzfeld, Germany). *Il*21^−/−^ mice on BL/6J background^77^ were kindly provided by Prof. Daniel Pinschewer (Department of Biomedicine, University of Basel). *Il21R*^−/−^ mice on BL/6J background^23^ and IL21^mCherry^ reporter mice^31^ were kindly provided by Prof. Manfred Kopf (Molecular Health Sciences, ETH Zurich). Experiments were performed with age- (6–8 weeks) and sex-matched animals of both genders and mice were randomly assigned to different treatment groups. Mice were housed under specific pathogen-free conditions in individually ventilated cages with food and water *ad libitum* and were regularly monitored for pathogens. Animal experiments were approved by the local experimental animal committee of the Canton of Bern and performed according to Swiss laws for animal protection (BE75/17, BE78/17, BE56/2020, BE59/2020, BE58/2021 and BE30/2021).

#### Patient samples

Blood samples, BM aspirates and serum from untreated, newly diagnosed AML patients at the Department of Hematology and Central Hematology Laboratory, Inselspital, Bern University Hospital and University of Bern, Switzerland, were obtained after written informed consent. BM from healthy donors was collected from orthopedic patients who underwent Vertebroplasty. Patient characteristics are listed in Tables S1 and S2. Analysis of blood, BM and serum samples was approved by the local ethical committee of the Canton of Bern, Switzerland (KEK 122/14 and 2019-01627).

#### Cell lines

THP-1 cells were purchased from ATCC, cultured in RPMI 1640 medium supplemented with 10% fetal calf serum (FCS), 100 U/ml penicillin, 100 μg/mL streptomycin and maintained in a humidified incubator at 37°C and 5% CO_2_.

### Method details

#### Antibodies for flow cytometry and cell sorting

##### Human

APC anti-CD34 (clone 561, 1:80), PE-Cy7 anti-CD38 (clone HIT2, 1:50), APC-Cy7 anti-CD4 (clone RPA-T4, 1:80), PerCP-Cy5.5 anti-CD8 (clone HIT8a, 1:100), Pacific Blue Annexin V (1:50) were purchased from BioLegend. V500 anti-CD45 (clone 2D1, 1:50) was purchased by BD Biosciences. PE anti-IL21R (clone REA233, 1:10) and PE anti-CD132 (clone REA313, 1:10), PE REA Control (S) human IgG1 isotype were purchased from Miltenyi. Differentiated cells were excluded by using biotin-conjugated antibodies against CD2 (clone RPA-2.10), CD14 (clone HCD14), CD16 (clone 3G8), CD19 (clone HIB19), CD56 (clone HCD56), CD235a (clone HIR2) (all 1:100; BioLegend), followed by staining with FITC-conjugated streptavidin (1:3000; BioLegend).

##### Mouse

APC anti-Ly-6C/G (Gr1-1) (clone RB6-8C5, 1:200), PE-Cy7 anti-CD11b (clone M1/70, 1:200), APC-Cy7 anti-CD19 (clone 6D5, 1:300), PerCP-Cy5.5 anti-Sca-1 (clone D7, 1:600), APC-Cy7 anti-CD117 (c-kit) (clone 2B8, 1:300), PE-Cy7 anti-CD16/32 (Fcγ) (clone 93, 1:400), BV650 anti-CD4 (clone RM4-5, 1:600), Alexa Fluor 700 anti-CD8 (clone 53-6.7, 1:800) were purchased from BioLegend. e506 Fixable Viability Dye (1:1000), eFluor450 anti-CD34 (clone RAM34, 1:100) were purchased from ThermoFisher Scientific. Lineage positive were excluded by using biotin-conjugated antibodies against CD19 (clone 6D5), CD3e (clone 145-2C11), Ly-6C/G (Gr1-1) (clone RB6-8C5), Ter119 (clone Ter-119) (all 1:300; BioLegend), followed by staining with V500-streptavidin (1:1000; BD Biosciences) or APC-streptavidin (1:3000; BioLegend).

Samples were acquired on a BD LSR Fortessa and sorting procedures were conducted using a BD FACS Aria III (both BD Biosciences). Data were analyzed using FlowJo software v.10.6 (TreeStar).

#### IL21 determination in human serum and mouse BM

Human IL21 protein levels in serum samples from newly diagnosed, untreated AML patients were determined by enzyme-linked immunosorbent assay (ELISA) using an IL21 Human ELISA kit (Biolegend), according to the manufacturer’s instructions.

To obtain murine BM supernatant, bones were flushed in 400 μL of phosphate-buffered saline (PBS) solution and supernatant was collected after pelleting the cells. Mouse IL21 protein levels in BM supernatant were determined using an IL21 Mouse ELISA kit (ThermoFisher Scientific).

#### Colony-forming assay

##### Human

3x10^3^ FACS-purified CD45^dim^SSC^lo^lin^−^CD34^+^ AML stem and progenitor cells from BM and PB of AML patients were cultured overnight in 96-well V-bottom plates (Corning) in Stem Span Medium (STEMCELL Technologies) supplemented with 100X Stem Span Cytokine Cocktail (STEMCELL Technologies), in the presence or absence of 100 pg/mL rh-IL21 (Sigma-Aldrich). The next day, cells were plated into semi-solid methylcellulose (MethoCult H4435 Enriched, STEMCELL Technologies or Enriched Human Methylcellulose, R&D Systems), with further addition of 100 pg/mL rh-IL21. For the second plating, total cells were collected from the methylcellulose and counted, followed by replating of 10-fold higher cell number into methylcellulose, without any treatment. Colonies number (≥30 cells/colony) was assessed by inverted light microscopy after 2 weeks for each round of plating.

For CAR T cell experiments, FACS-purified CD45^dim^SSC^lo^lin^−^CD34^+^ stem and progenitor cells from the BM of AML patients were cultured overnight in Stem Span Medium (STEMCELL Technologies) supplemented with 100X Stem Span Cytokine Cocktail (STEMCELL Technologies) with of 100 pg/mL recombinant human (rh)-IL21 (Sigma-Aldrich) in presence and absence of CAR T cells targeting CD70 at an effector:target ratio of 1:5. The next day, cells were plated into semi-solid methylcellulose (MethoCult H4435 Enriched, STEMCELL Technologies or Enriched Human Methylcellulose, R&D Systems), and colony numbers were assessed 14 days later.

##### Mouse

For assessment of colony forming units, 5x10^4^ total bone marrow cells from AML mice were plated into semi-solid methylcellulose. Number of MLL-AF9-GFP^+^ or MLL-ENL- YFP^+^ colonies (≥30 cells/colony) was assessed after 7 days by fluorescence microscopy.

For testing the effect of recombinant mouse IL21 on LSCs colony-forming capacity, 10^3^ FACS-purified GFP^+^lin^−^Sca-1^−^c-kit^high^CD34^+^Fcγ^+^ GMPs from AML mice were cultured overnight in 96-well V-bottom plates (Corning) in RPMI 1640 medium supplemented with 10% FCS, 1% L-glutamine, 1% penicillin/streptomycin, 100 ng/mL recombinant mouse (rm)-SCF (Miltenyi) and 20 ng/mL rm-TPO (BioLegend), in the presence or absence of 300 pg/mL rm-IL21 (R&D Systems). The next day, cells were plated in semi-solid methylcellulose. Number of colonies was assessed by inverted light microscopy after 7 days.

In both assays, the semi-solid methylcellulose used was MethoCult M3134 medium (STEMCELL Technologies), supplemented with 15% FCS, 20% BIT (50 mg/mL BSA in IMDM, 1.44 U/ml rh-insulin [Actrapid; Novo Nordisk], and 250 ng/mL human holo transferrin [Prospec]), 100 μM 2-mercaptoethanol, 100 U/ml penicillin, 100 μg/mL streptomycin, 2 mM L-glutamine, and 50 ng/mL rm-SCF, 10 ng/mL rm-IL3, 10 ng/mL rm-IL6, and 50 ng/mL rm-Flt3-ligand (all Miltenyi).

#### Short-term LSPCs liquid culture

FACS-purified CD45^dim^SSC^lo^lin^−^CD34^+^ AML stem and progenitor cells from BM and PB of AML patients were cultured for 72 h in 96-well V-bottom plates (Corning) in StemSpan Medium (STEMCELL Technologies) supplemented with 100X StemSpan Cytokine Cocktail (STEMCELL Technologies), in the presence or absence of 100 pg/mL rh-IL21 (Sigma-Aldrich) and/or 1 nM cytarabine (Cytosine β-D-arabinofuranoside, Sigma-Aldrich). Cells were counted after 72 h to determine cell growth and stained with AnnexinV to assess viability.

#### Murine syngeneic AML models

MLL-AF9 AML was induced by transducing FACS-purified LSKs with the GFP-MLL-AF9 retroviral construct^26^ by spin infection on two consecutive days. 5x10^4^ cells were injected into the tail vein of non-irradiated syngeneic recipients.

MLL-ENL AML was induced by retroviral transduction of FACS-purified LSKs with the YFP-MLL-ENL oncogene,^78^ with spin infection on two consecutive days. 2.5x10^4^ cells were injected into the tail vein of sublethally irradiated (4.5 Gy) syngeneic recipients.

#### Murine patient-derived xenograft AML model

Xenotransplantations were performed as previously described.^52^ In brief, NSG-S mice were sublethally irradiated (1.5 Gy) on the day before injection. 10^6^ FACS-purified CD45^dimS^SC^lo^ blasts from the BM of newly diagnosed AML patients (AML 182 and AML 185, Table 1) were injected i.v. into the tail vein. Starting 10 days after transplantation, mice were randomized, and 20 μg rh-IL21 or control vehicle was administered i.p. daily in 5 days on-2 days off regimen. Mice were monitored daily for signs of morbidity (significant weight loss, failure to groom, abnormal gait, and posture) and euthanized when terminally ill.

#### LSPCs analysis

##### Human

AML stem and progenitor cells from BM and PB primary samples for phenotypical analysis and/or FACS-purification were defined as CD45^dim^SSC^lo^lin^−^CD34^+^ according to several publications.^36^^,^^79^^,^^80^

##### Mouse

L-GMPs in BM and spleen of AML mice were for phenotypical analysis and/or FACS-purification were defined as GFP/YFP^+^lin^−^Sca-1^−^c-kit^high^CD34^+^Fcγ^+^ cells according to Somervaille and Cleary, 2006.^26^

#### Numb staining and ImageStream analysis

FACS-sorted L-GMPs were fixed by incubation in 4% paraformaldehyde (PFA), followed by permeabilization with 1X wash buffer (Dako Wash, Agilent Technologies) and blocking with 10% normal rabbit serum and donkey serum (Invitrogen) in Dako Wash. Numb and α-tubulin staining was performed overnight at 4°C (with goat anti-Numb polyclonal ab, 1:20; and mouse anti-tubulin mAb, 1:400; both Abcam) in diluent (Dako antibody diluent, Agilent Technologies). Cells were then incubated with the secondary antibody (AlexaFluor568-conjugated donkey-anti goat ab, 1:400, and AlexaFluor647-conjugated rabbit-anti mouse ab, 1:2000; both Abcam) for 1 h at room temperature. DAPI (Roche) was used to stain for DNA. Samples were acquired using an ImageStreamxMkII imaging flow cytometer (Merck) and dividing cells were analyzed using INSPIRE and IDEAS Software. A difference in Numb staining of at least 1.8-fold was defined as asymmetric cell division as described by Zimdahl et al., 2014.^81^

#### brdU staining

1 mg brdU solution (BD Biosciences) was injected intraperitoneally 48 h prior to sacrificing and analyzing the mice. brdU staining was performed using the brdU APC kit (BD Biosciences), according to the manufacturer’s instructions. Briefly, after cell surface markers staining, whole BM cells were fixed and permeabilized by incubation in BD Cytofix/Cytoperm. Next, cells were incubated with 300 μg/mL DNase for 1 h at 37°C to expose incorporated brdU and then stained with an APC anti-brdU antibody (1:50, BD Biosciences). 1X BD Perm/Wash in ddH_2_O was used as a staining and washing buffer. Samples were acquired on an LSRII (BD Biosciences) flow cytometer.

#### NF-kB staining

Whole BM cells were fixed by incubation in 4% PFA for 15 min, followed by permeabilization in ice-cold 90% methanol in 1X PBS. The following antibodies were used for intracellular staining respectively of IKBα and phosphoIKBα: rabbit mAb anti-IKBα (44D4) (1:100, Cell Signaling) and rabbit mAb anti-phospho-IKBα (Ser32) (14D4) (1:100, Cell Signaling), followed by staining with an AlexaFluor647-conjugated anti-rabbit IgG F(ab')2 Fragment (1:800, Cell Signaling). 0.5% BSA in 1X PBS was used as a staining and washing buffer. Samples were acquired on an LSRII (BD Biosciences) flow cytometer.

#### p38-MAPK staining

Intracellular p38-MAPK staining was performed for FACS-sorted L-GMPs from BM of BL/6, *Il21*^*−/−*^, *Il21R*^*−/−*^ and *Il21R*^*+/−*^ AML mice and for THP-1 AML cells, which were first treated with 1 ng/mL rh-IL21 for 72 h or left untreated. After staining with eFluor450 Fixable Viability Dye (1:1000; ThermoFisher Scientific), cells were fixed with Cytofix/Cytoperm (BD Bioscience) as per manufacturer protocol. Subsequently cells were washed with PBS and permeabilized with True-Phos Perm Buffer (BioLegend) according to the manufacturer’s protocol. Cells were washed with PBS and intracellularly stained with PE anti-phospho-p38-MAPK (Thr180/Tyr182; 1:20; BioLegend) ab or PE mouse isotype control IgG1, κ (1:20; BioLegend) for 30 min at room temperature. Cells were acquired on an LSRII (BD Biosciences) flow cytometer and analyzed by Kaluza Analysis software.

#### ROS and mitochondrial dyes staining

Staining with CellROX, MitoTracker and TMRM was performed on whole BM cells, after cell surface markers staining. For staining, cells were incubated for 30 min at 37°C with, respectively, 5 μM CellROX, 25 nm MitoTracker, 10 nm TMRM in RPMI 1640 medium. 50 μM of verapamil hydrochloride was used to inhibit dyes efflux from mitochondria. Cells were washed three times with PBS to remove the excess of dyes and were acquired on an LSRII (BD Biosciences) flow cytometer.

#### Cell culture with p38 MAPK inhibitor

##### THP-1 cell line

10^5^ human THP-1 AML cells were pretreated for 30 min with vehicle or 10 nm/mL of the p38 MAPK inhibitor SB203580 and were then cultured for 72 h in the presence or absence of 1 ng/mL rh-IL21 in technical triplicates. After 72 h of culture, cell numbers were assessed by Trypan-blue exclusion and intracellular ROS and p38-MAPK phosphorylation by flow cytometry.

##### Murine L-GMPs

10^3^ FACS-purified GFP^+^lin^−^Sca-1^−^c-kit^high^CD34^+^Fcγ^+^ GMPs from AML mice were pretreated for 30 min with vehicle or 10 nm/mL of the p38 MAPK inhibitor SB203580 and were then cultured overnight in 96-well V-bottom plates (Corning) in RPMI 1640 medium supplemented with 10% FCS, 1% L-glutamine, 1% penicillin/streptomycin, 100 ng/mL recombinant mouse (rm)-SCF (Miltenyi) and 20 ng/mL rm-TPO (BioLegend), in the presence or absence of 300 pg/mL rm-IL21 (R&D Systems). The next day, cells were plated in semi-solid methylcellulose (described above). Number of colonies was assessed by inverted light microscopy after 7 days.

#### Quantitative Reverse Transcription PCR analysis of gene expression

For quantitative Reverse Transcription PCR (qRT-PCR), total RNA was extracted from FACS-sorted cell populations using the Quick-RNA MiniPrep kit (Zymo Research) according to the manufacturer’s instructions. Total RNA was reverse-transcribed using 2.5x10-4 U/μl hexanucleotide mix (Roche), 0.4mM deoxynucleotide mix (Sigma-Aldrich), 1.25 U/μl RNAsin (Promega) and 4 U/μl reverse transcriptase (Promega). 2 μL of cDNA were used for Real-Time PCR with self-designed primers and SYBR green reaction (Roche). qRT-PCR reactions were performed in duplicates including non-template controls on a QuantStudio 3 Real-Time PCR system (Applied Biosystems). Expression levels of analyzed genes relative to a reference gene (ACTB or Actb) were calculated using the comparative Ct method (also referred to as the 2^−ΔΔCt^ method).^82^ The following primer pairs were used to determine mRNA expression of respective genes:

*Il21*, FW: CACATAGCTAAATGCCCTTCC, RV: CCTCAGGAATCTTCGGGTC;

*Actb*, FW: AGATGACCCAGATCATGTTTGAG, RV: GTACGACCAGAGGCATACAG;

*IL21*, FW: TTATGTGAATGACTTGGTCCCT, RV: CTGTATTTGCTGACTTTAGTTGGG;

*IL21R*, FW: TCATCTTTCAGACCCAGTCAG, RV: CATATCTTCTTCCATAGCCTCCAC;

*ACTB*, FW: GCACCACACCTTCTACAATGAG, RV: GGTCTCAAACATGATCTGGGTC.

#### High-throughput transcriptome analysis using next generation RNA sequencing

Total RNA was extracted from FACS-sorted L-GMPs from BL/6 and *Il21*^−/−^ AML mice using the RNeasy Micro Kit (Qiagen). RNA purity was checked using the NanoPhotometer spectrophotometer (Implen). RNA integrity and quantity were assessed using the RNA Nano 6000 Assay Kit of the Bioanalyzer 2100 system (Agilent Technologies). A total amount of 1 μg RNA per sample was used as input material for the libraries preparations. Sequencing libraries were generated using NEBNext UltraTM RNA Library Prep Kit for Illumina (New England Biolabs Inc.) and index codes were added to attribute sequences to each sample. Library quality was assessed on the Agilent Bioanalyzer 2100 system (Agilent Technologies). The clustering of the index-coded samples was performed on a cBot Cluster Generation System using PE Cluster Kit cBot-HS (Illumina). After cluster generation, the libraries were sequenced on a Nova 6000 Illumina platform and paired-end reads were generated.

#### RNA-seq analysis and gene set enrichment analysis

The quality of the RNA-seq data was assessed using fastqc v. 0.11.9 (http://www.bioinformatics.babraham.ac.uk/projects/fastqc/) and RSeQC v. 4.0.0.^72^ The reads were mapped to the GRCm38 reference genome using HiSat2 v. 2.2.1.^73^ FeatureCounts v. 2.0.1^74^ were used to count the number of reads overlapping with each gene as specified in the genome annotation (Ensembl build 100 and Homo_sapiens.GRCh38.104).

The R Bioconductor package DESeq2 v1.32.0^75^ was used to test for differential gene expression between the experimental groups. TopGo v2.44.0 was used to identify gene ontology terms containing unusually many differentially expressed genes. An interactive Shiny application was set up to facilitate the exploration and visualization of the RNA-seq results.

Gene set enrichment analysis (GSEA) was run with ClusterProfiler v4.0.2,^76^ using gene sets from the Broad Institute’s Molecular Signatures Database (MSigDB Hallmarks collection, available at gsea-msigdb.org), Pathcards database (available at https://pathcards.genecards.org/) and KEGG database (available at https://www.genome.jp/kegg/pathway.html). Further visualization was performed using R version 4.1.0.

#### ELISPOT

BM CD4^+^ and CD8^+^ T cells and LSPCs were FACS-purified and human IL21 secreting cells were analyzed via ELISPOT based on the manufacturer’s description (Mabtech).

#### CAR construct generation

The backbone of a CD19-CAR plasmid^83^ was kindly provided by Michele Bernasconi (Addgene #200671) and used to generate the CD70-CAR plasmid. The CD70 VH and VL chains of the CD70 CAR were taken from the CD70 Cusatuzumab antibody^38^ and associated via a polylinker.

#### Generation of CAR-expressing lentivirus

Lentivirus encoding the CD70 CAR construct was produced via third generation plasmid transfection of HEK-293T cells. HEK cells were transfected using Lipofectamine LTX, respective CAR plasmids and 3 helper plasmids (Addgene: pMDLg/pRRE #12259, pRSV-Rev #12253, pCMV-VSV-g #8454). Viral supernatant was harvested 48 h post-transfection and concentrated with PEG-it reagent (VWR). Healthy donor-derived T cells were isolated from peripheral blood mononuclear cells (PBMCs) and activated for 48 h using TransAct beads (Miltenyi). Upon activation, T cells were transduced with CD70 CAR viral particles with a MOI of 1, in presence of polybrene (10 μg/mL) at 32°C 800g for 90 min. Transduction efficiency was measured 7 days post transduction by flow cytometry (Fortessa) measuring eGFP expression. CAR T cells were expanded and maintained in TexMACS (Miltenyi) supplemented with 10 ng/mL IL7 and 10 ng/mL IL15 (Miltenyi).

### Quantification and statistical analysis

All flow cytometry, *in vitro* and *in vivo* data were analyzed and plotted using GraphPad Prism software v9.0 (GraphPad). Bars and error bars indicate means, standard errors of mean and standard deviations of the indicated number of independent biological replicates. Two-tailed Student’s t test, Mann-Whitney test, Pearson r test, one-way-ANOVA followed by Tukey’s post-test and two-way ANOVA followed by Sidak’s post-test were used as indicated in the figures legends. Significance of differences in Kaplan-Meier survival curves was determined using the log rank test (two-tailed). LSC frequencies with 95% confidence intervals (CI) were estimated with ELDA software (http://bioinf.wehi.edu.au/software/elda/) and significant differences in LSC frequency were calculated by χ2 test in limiting dilution assay.^27^

*p* < 0.05 was considered significant. Details on the quantification, normalization and statistical tests used in every experiment can be found in the corresponding figure legend. n represents the number of independent replicates in each experiment.

## Supplemental information

Document S1. Figures S1–S7 and Table S2

Table S1. Characteristics of patients with AML, related to Figure 5

Document S2. Article plus supplemental information
