# Supplementary material for: IL-21/IL-21R signaling renders acute myeloid leukemia stem cells more susceptible to cytarabine treatment and CAR T cell therapy
Source: Cell Rep Med. 2024 Nov 12;5(11):101826. doi: 10.1016/j.xcrm.2024.101826 (PMC11604404; doi:10.1016/j.xcrm.2024.101826)
Supplement: Document S1. Figures S1–S7 and Table S2 [file mmc1.pdf]

Cell Reports Medicine, Volume 5

Supplemental information

IL-21/IL-21R signaling renders acute myeloid leukemia stem cells more susceptible to cytarabine treatment and CAR T cell therapy

Viviana Rubino, Michelle Hüppi, Sabine Höpner, Luigi Tortola, Noah Schnüriger, Hugo Legenne, Lea Taylor, Svenja Voggensperger, Irene Keller, Remy Bruggman, Marie-Noëlle Kronig, Ulrike Bacher, Manfred Kopf, Adrian F. Ochsenbein, and Carsten Riether

SUPPLEMENTAL INFORMATION

Figure S1

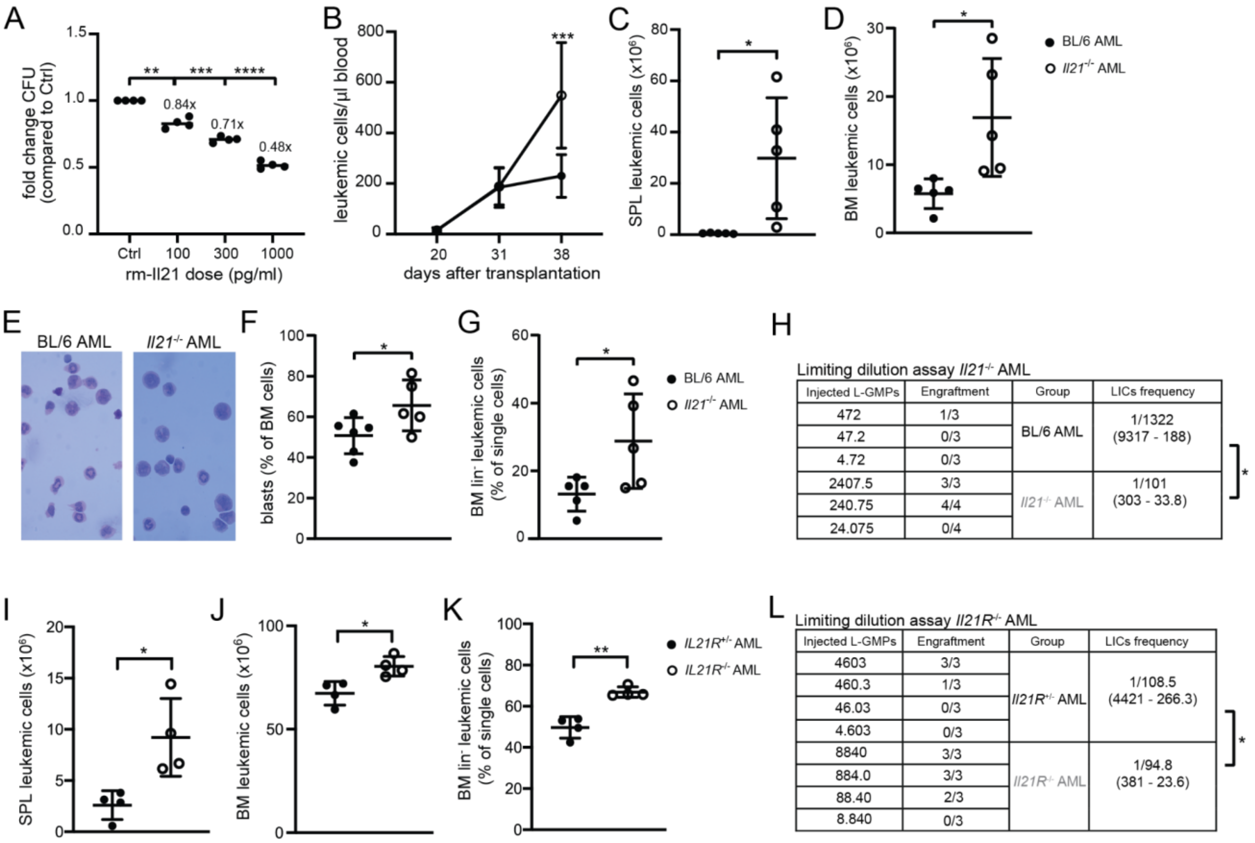

Figure S1. An IL21-deficient microenvironment, as well as IL21R deficiency on leukemia-initiating cells result in increased AML burden and accumulation of primitive leukemic cells in BM of mice. Related to Figure 1. (A) Fold change colony-forming units from FACS-sorted L-GMPs cultured in methylcellulose for seven days in the presence of increasing doses of rm-Il21. L-GMPs were FACS-sorted from n = 4 BL/6 AML mice. Each dot represents the mean of three technical replicates. Statistics were determined by a paired Student's *t* test (vs. ctrl). **(B)** Number of MLL-AF9-GFP$^+$ leukemic cells on days 20, 31 and 38 in the blood of BL/6 and *Il21*$^{-/-}$ AML mice (n = 5 mice/group). Data are displayed as mean ± SD. Statistics were determined by two-way ANOVA followed by Sidak's multiple comparisons test. **(C, D)** MLL-AF9-GFP$^+$Gr1$^+$Cd11b$^+$ cells in the spleen **(C)** and in the BM **(D)** of BL/6 and *Il21*$^{-/-}$ AML mice (n = 5 mice/group). Data are displayed as mean ± SD. Statistics were determined by Student's t test. **(E)** Representative H&E-stained cytospin preparations of BM, **(F)** quantification of blasts percentage by microscopic evaluation of cell morphology and **(G)** percentage of lineage negative MLL-AF9-GFP$^+$ leukemic cells in BM of BL/6 and *Il21*$^{-/-}$ AML mice (n = 5 mice/group). **(B – G)** One representative of four independent experiments is shown. **(H)** Recalculation of leukemia-initiating cell (LIC) frequency from the ELDA assay shown in Fig. 1I, after normalizing injected cells for the actual number of immunophenotypically-defined L-GMPs (MLL-AF9-GFP$^+$lin$^-$Sca-1$^-$c-kit^{high}CD34$^+$Fcγ$^+$) transferred. **(I, J)** MLL-AF9-GFP$^+$Gr1$^+$Cd11b$^+$ cells in the spleen **(I)** and in the BM **(J)** and **(K)** percentage of lineage negative MLL-AF9-GFP$^+$ leukemic cells in BM of *Il21R*$^{+/-}$ and *Il21R*$^{-/-}$ AML mice (n = 4 mice/group). **(I – K)** One representative of two independent experiments is shown. Data are displayed as mean ± SD. Statistics were determined by Student's t test. **(L)** Recalculation of leukemia-initiating cell (LIC) frequency from the ELDA assay shown in Fig. 1N, after normalizing injected cells for the actual number of immunophenotypically-defined L-GMPs transferred. *, P < 0.05; **, P < 0.01, ***, P < 0.001, ****, P < 0.0001. Abbreviations: SPL, spleen: BM, bone marrow; Lin, lineage; ELDA, extreme limiting dilution analysis; L-GMPs, leukemic granulocyte-macrophage progenitors; LIC, leukemia-initiating cell.

Figure S2

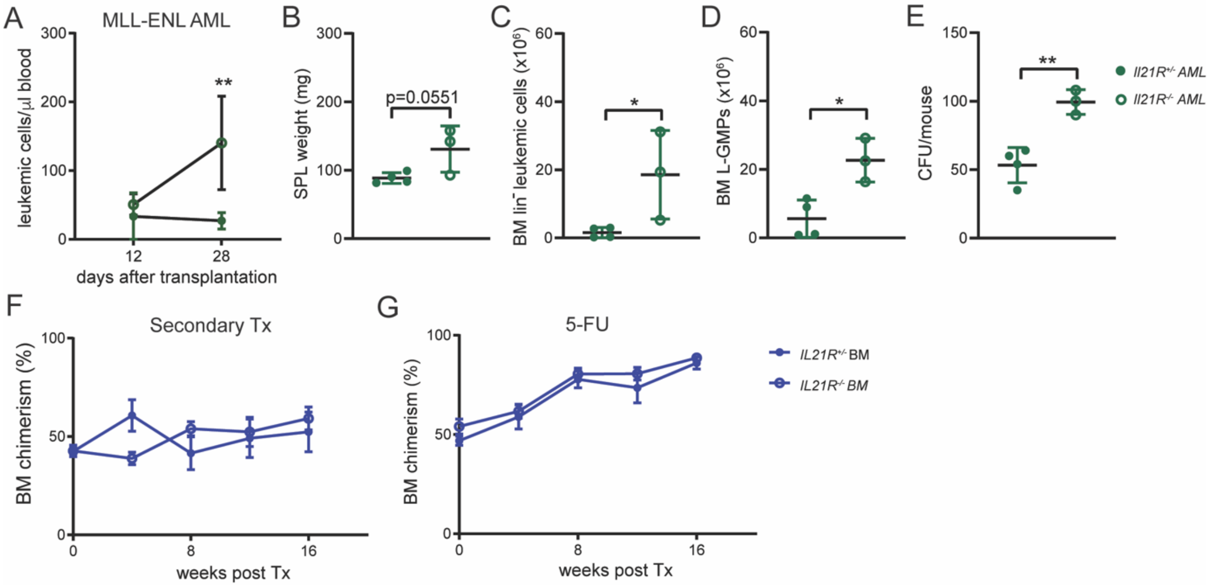

Figure S2. IL21R deficiency on leukemia-initiating cells results in faster disease development and accumulation of primitive leukemic cells in an MLL-ENL-driven AML model. IL21R deficiency on normal hematopoietic stem cell does not affect their repopulating capacity in steady-state and stress-induced hematopoiesis. Related to Figure 1. (A - D) $2.5x10^4$ MLL-ENL-YFP-transduced LSKs from the BM of *Il21R*$^{-/-}$ and *Il21R*$^{+/-}$ mice were injected intravenously into sublethally-irradiated (4.5 Gy) *Il21R*$^{+/-}$ recipients (*Il21R*$^{-/-}$ AML and *Il21R*$^{+/-}$ AML, respectively). Mice were sacrificed 30 days after leukemia transplantation and BM and spleen were analyzed (n = 3-4 mice/group). One representative of two independent experiments is shown. **(A)** Number of MLL-ENL-YFP^{+} leukemic cells on days 12 and 28 in the blood of *Il21R*$^{-/-}$ and *Il21R*$^{+/-}$ AML mice. Data are displayed as mean ± SD. Statistics were determined by two-way ANOVA followed by Sidak's multiple comparisons test. **(B)** Spleen size, **(C)** number of lineage negative MLL-ENL-YFP^{+} leukemic cells and **(D)** number of L-GMPs in BM of *Il21R*$^{-/-}$ AML and *Il21R*$^{+/-}$ AML mice. Data are displayed as mean ± SD. Statistics were determined by Student's *t* test. **(E)** Colony forming units per mouse. $5x10^4$ BM cells were plated into methylcellulose and YFP^{+} colonies were enumerated seven days later by inverted fluorescence microscopy. Data are displayed as mean ± SD. Statistics were determined by Student's *t* test. **(F)** BM reconstitution after transplantation of *Il21R*$^{-/-}$ and *Il21R*$^{+/-}$ donor cells into lethally irradiated (2 x 6.5 Gy) congenic secondary recipients. BM chimerism measured at week 4, 8, 12 and 16 post transplantation. Data are displayed as mean ± SEM. Statistics were determined by two-way ANOVA followed by Sidak's multiple comparisons test. **(G)** BM reconstitution after 5-FU treatment followed by transplantation of *Il21R*$^{-/-}$ and *Il21R*$^{+/-}$ donor cells into lethally irradiated (2 x 6.5 Gy) congenic secondary recipients. BM chimerism measured at week 4, 8, 12 and 16 post transplantation. Data are displayed as mean ± SEM. Statistics were determined by two-way ANOVA followed by Sidak's multiple comparisons test. *, $P < 0.05$; **, $P < 0.01$. Abbreviations: SPL, spleen; L-GMPs, leukemic granulocyte-macrophage progenitors; CFU, colony-forming units; Tx, transplantation; 5-FU, 5-fluorouracil.

Figure S3

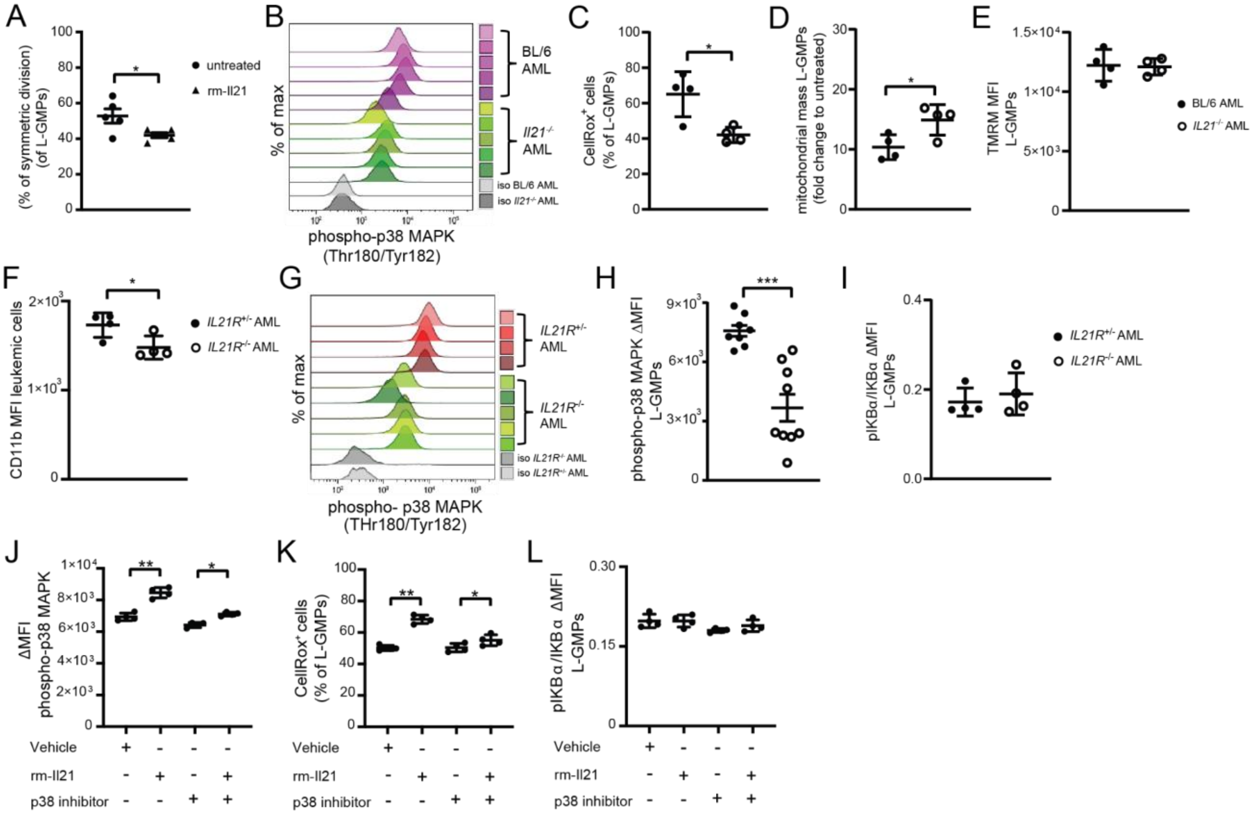

Figure S3. Il21/Il21R signaling regulates cell L-GMPs in AML by inducing differentiation, accumulation of ROS and activation of the p38-MAPK signaling pathway. Related to Figure 2. (**A)** FACS-sorted L-GMPs from the BM of BL/6 AML mice were cultured for 48h in the presence or absence of 300 pg/ml rm-Il21. Cells in symmetric division were quantified by Numb staining and ImageStream® analysis. Statistics were determined by Student's *t* test. **(B)** Histograms showing phosphorylation of p38 MAPK (phospho-p38 MAPK) in L-GMPs from BM of BL/6 and *Il21*$^{-/-}$ AML mice (n = 5 mice/group). **(C)** Intracellular reactive oxygen species measured as frequency of L-GMPs positive to CellRox™ staining, (**D**) mitochondrial mass determined by MitoTracker™ staining and (**E**) mitochondrial membrane potential determined by TMRM™ staining of L-GMPs from BM of BL/6 and *Il21*$^{-/-}$ AML mice. One representative of two independent experiment is shown (n = 4 mice/group). Data are displayed as mean ± SD. Statistics were determined by Student's *t* test. **(F)** CD11b mean fluorescence intensity of MLL-AF9-GFP^{+} leukemic cells from BM of *Il21R*$^{+/-}$ and *Il21R*$^{-/-}$ AML mice (n = 4 mice/group). Data are displayed as mean ± SD. Statistics were determined by Student's *t* test. **(G)** Histograms showing phosphorylation of p38 MAPK (phospho-p38 MAPK) and **(H)** geometric mean fluorescence intensity (MFI) quotient of phospo-p38 MAPK staining versus its isotype control on L-GMPs from BM of *Il21R*$^{+/-}$ and *Il21R*$^{-/-}$ AML mice. Two pooled independent experiments are shown (n = 4- 5 mice/group). Data are displayed as mean ± SD. Statistics were determined by Student's *t* test. **(I)** NF-kB pathway activation measured as ratio between protein expression of IκBα and its phosphorylated form pIκBα in L-GMPs from BM of *Il21R*$^{+/-}$ and *Il21R*$^{-/-}$ AML mice (n = 4 mice/group). Data are displayed as mean ± SD. Statistics were determined by Student's *t* test. **(J – L)** FACS-purified L-GMPs from BL/6 AML mice were pre-treated with 10 nm/ml of the p38 MAPK inhibitor SB203580 or vehicle, prior to overnight culture in in the presence or absence of 300 pg/ml rm-IL21. MFI of phospo-p38 MAPK staining versus its isotype control **(J)**, CellRox^{+} cells **(K)** and ratio between protein expression of IκBα and pIκBα **(L)** were measured. Statistics were determined by Student's *t* test. *, $P < 0.05$; **, $P < 0.01$; ***, $P < 0.001$. Abbreviations: L-GMPs, leukemic granulocyte-macrophage progenitors; TMRM, tetra-methylrhodamine, methyl ester.

Figure S4

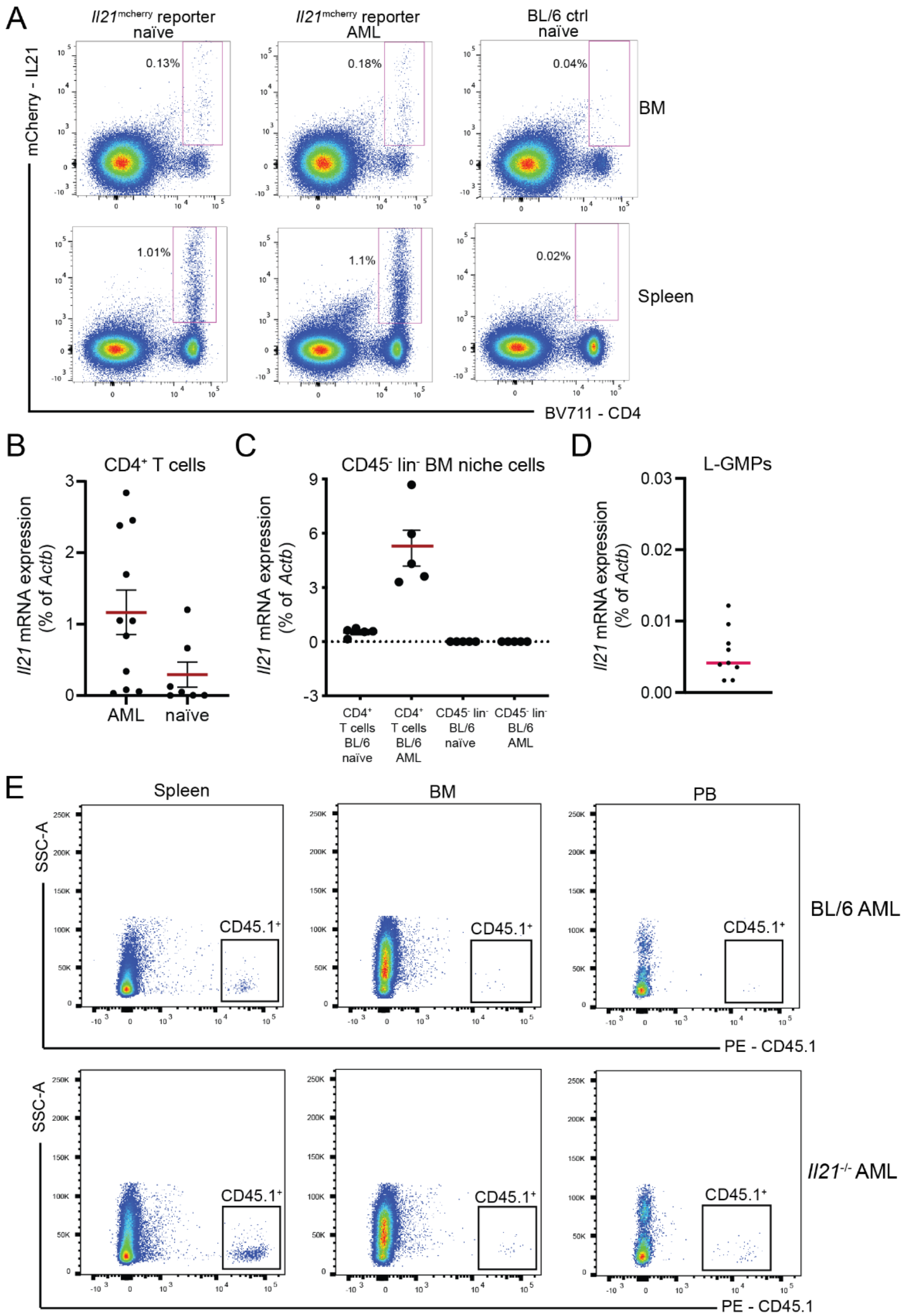

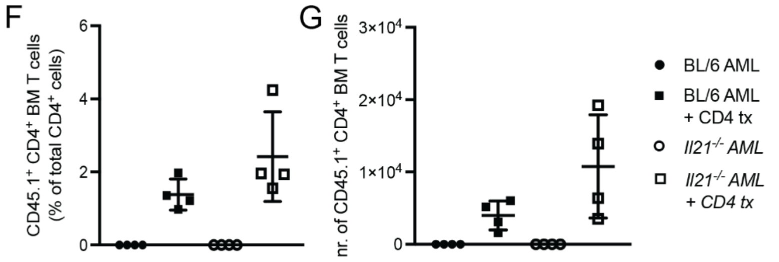

H

	Injected L-GMPs	Engraftment	LIC frequency
BL/6 AML	$4.4x10^3$<br>$4.4x10^2$<br>$4.4x10^1$	4/4<br>2/4<br>0/4	1/712<br>(2526 - 198)
BL/6 AML +CD4+	$5x10^3$<br>$5x10^2$<br>$5x10^1$	4/4<br>3/4<br>0/4	1/444<br>(1408 - 140)
Il21-/- AML	$7.3x10^3$<br>$7.3x10^2$<br>$7.3x10^1$	4/4<br>4/4<br>2/4	1/104<br>(400 - 27)
Il21-/- AML +CD4+	$2.7x10^3$<br>$2.7x10^2$<br>$2.7x10^1$	4/4<br>1/4<br>0/4	1/753<br>(2437 - 233)

ns
*
ns
ns
ns
*

Figure S4. CD4$^+$ T cells from AML mice express *Il21*, unlike BM stromal cells and CD4$^+$ T cells from naïve BL/6 mice. Adoptively transferred CD4$^+$ T cells can be detected by flow cytometry in spleen, BM and peripheral blood of AML mice. Related to Figure 3. (A) Representative FACS plots of CD4$^+$ T cells in the BM and spleen of respectively IL21^{mCherry} naïve and AML reporter mice and naïve BL/6 mice. **(B)** *Il21* mRNA expression measured by qRT-PCR in FACS-sorted CD4$^+$ T cells from the BM of AML (n = 11) and naïve (n = 7) mice. Data are displayed as mean ± SEM. **(C)** *Il21* mRNA expression measured by qRT-PCR in FACS-sorted CD4$^+$ T cells and CD45$^-$lin$^-$ stromal cells from the BM of AML (n = 5) and naïve (n = 5) mice. Data are displayed as mean ± SEM. **(D)** *Il21* mRNA expression (qRT-PCR) in FACS-sorted L-GMPs from the BM of BL/6 AML mice thirty-five days after leukemia transplantation (n = 9). Red bar indicates the mean. **(E)** Representative FACS plots of adoptively transferred CD45.1$^+$ cells (pre-gated on single cells) detected by flow cytometry 36 days after the transfer, in spleen, BM and PB of BL/6 and *IL21$^{-/-}$* AML mice. **(F, G)** Quantification of adoptively transferred CD45.1$^+$CD4$^+$ T cells detected by flow cytometry 36 days after the transfer, the BM of BL/6 and *IL21$^{-/-}$* AML mice. CD45.1$^+$CD4$^+$ T cells frequency of total CD4$^+$ T cells **(F)** and absolute number of CD45.1$^+$CD4$^+$ T cells **(G)** are shown. **(H)** Recalculation of leukemia-initiating cell (LIC) frequency from the ELDA assay shown in Fig. 3I, after normalizing injected cells for the actual number of immunophenotypically-defined L-GMPs (MLL-AF9-GFP$^+$lin$^-$Sca-1$^-$c-kit^{high}CD34$^+$Fcγ$^+$) transferred. *, $P < 0.05$; Abbreviations: PB, peripheral blood; lin, lineage.

Figure S5

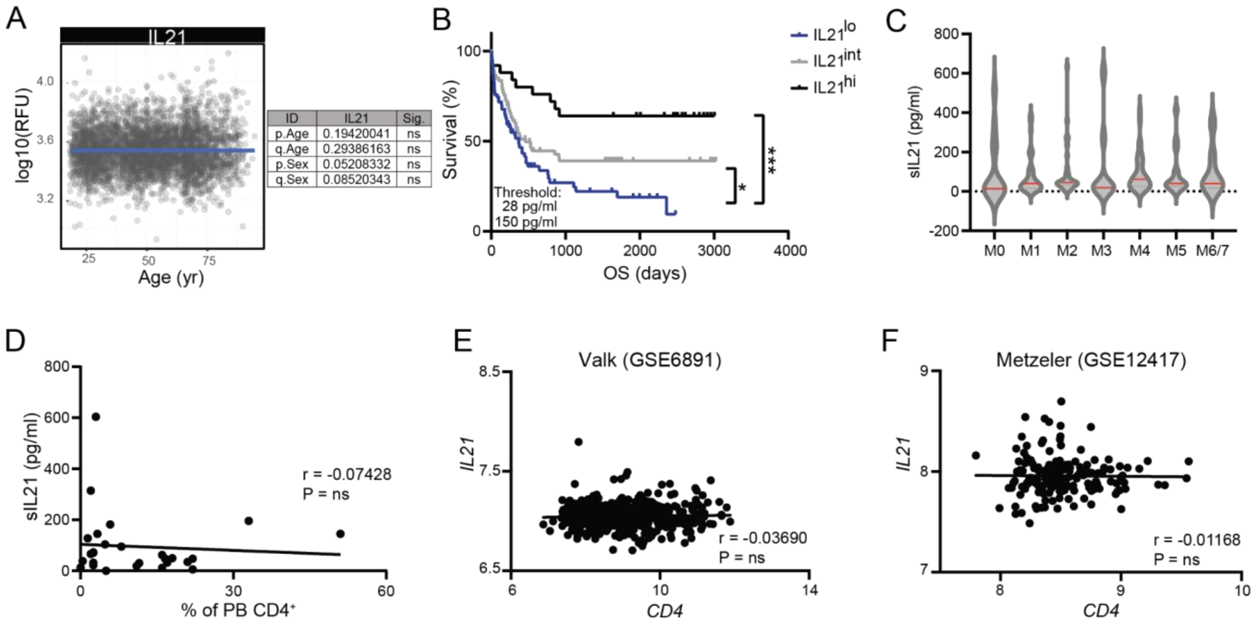

Figure S5. sIL21 levels are not altered with age in healthy individuals and do not correlate with frequencies of CD4$^+$ T cells in peripheral blood of AML patients. Related to Figure 4. (A) A publicly available plasma proteome dataset (human INTERVAL and LonGenity dataset, accession number EGAS00001002555) was analyzed for IL21 expression across lifespan (n = 4263 individuals). Statistics were determined with an age- and sex- adjusted linear model as described in Lehallier et al., 2019. **(B)** Kaplan-Meier survival curves of the entire AML patients' cohort (n = 193) divided into three groups at the sIL21 thresholds of 28 pg/ml and 150 pg/ml. Statistics were determined by log-rank test. **(C)** sIL21 levels in patients of the entire cohort stratified accordingly to their FAB classification at diagnosis. **(D)** sIL21 levels were correlated with the frequency of CD4$^+$ T cells in the peripheral blood of newly diagnosed AML patients (n = 27) determined by flow cytometry. Statistics were determined by Pearson r test. **(E, F)** *IL21* mRNA expression levels were correlated to *CD4* mRNA expression levels in the publicly available **(E)** Valk dataset (accession number GSE6891) and **(F)** Metzeler dataset (accession number GSE12417). Statistics were determined by Pearson r test. *, $P < 0.05$; ***, $P < 0.001$. Abbreviations: OS, overall survival; yr, years; PB, peripheral blood.

Figure S6

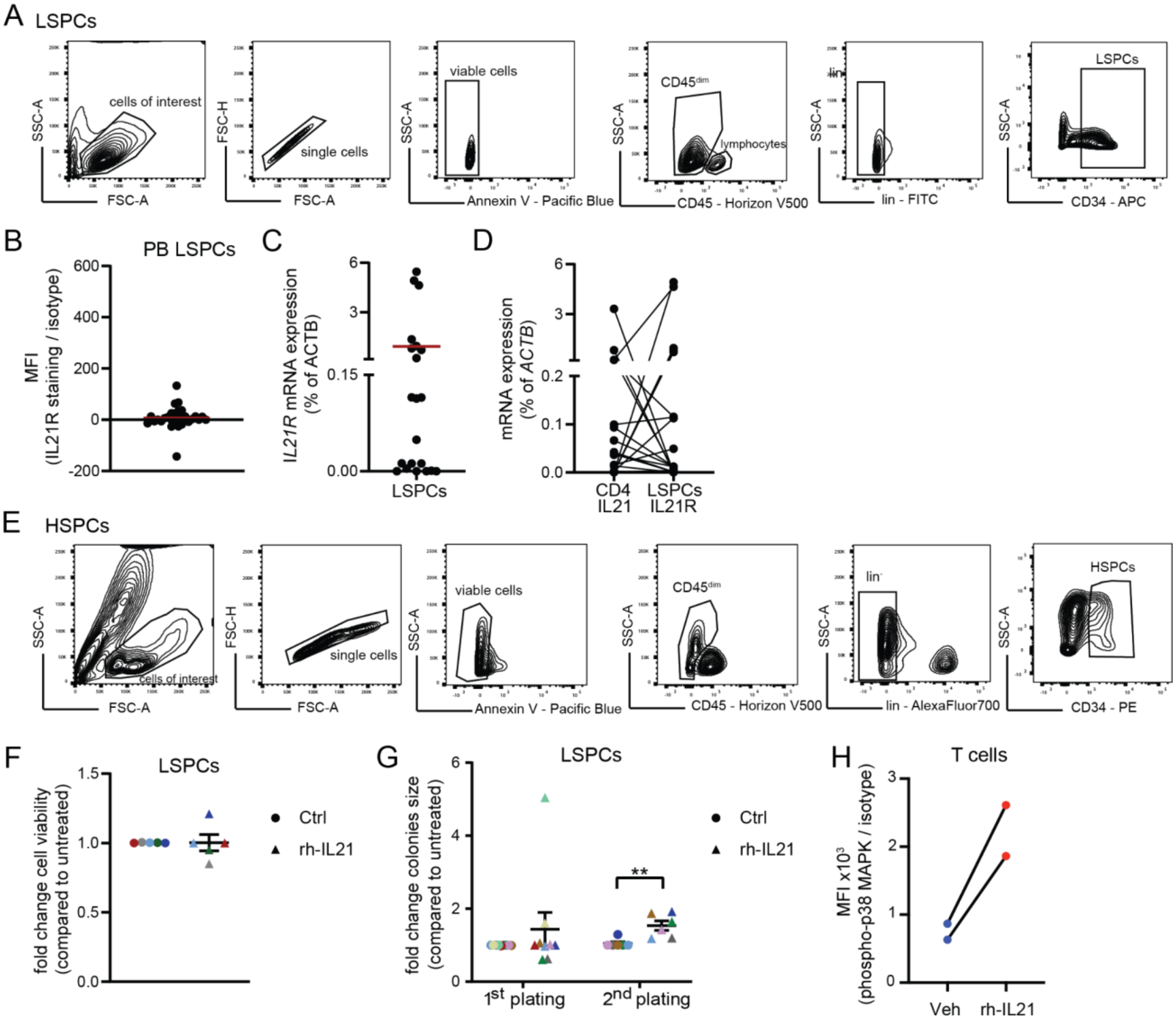

Figure S6. FACS gating strategy and IL21R expression on LSPCs in peripheral blood. Related to Figure 5. (A) Gating strategy to identify CD45^{dim} SSC^{lo} lin^{-}CD34^{+} AML stem and progenitor cells in BM samples from newly diagnosed AML patients. **(B)** Mean fluorescence intensity (MFI) quotient of IL21R staining versus its isotype control on LSPCs (n = 30) from blood samples of newly diagnosed AML patients. Red bar indicates the mean**. (C)** *IL21R* mRNA expression (qRT-PCR) in FACS-sorted LSPCs from newly diagnosed AML patients (n = 21). Red bar indicates the mean. **(D)** IL21 and IL21R mRNA expression (qRT-PCR) in paired CD4^{+} T cells and LSPCs FACS-sorted from newly diagnosed AML patients (n = 21). **(E)** Gating strategy to identify CD45^{dim} SSC^{lo}lin^{-}CD34^{+} stem and progenitor cells in BM samples of healthy controls who underwent BM biopsy for reasons other than leukemia. **(F)** Viability of n = 5 FACS-sorted LSPCs cultured in vitro for 72 h in the presence or absence of 100 pg/ml rh-IL21. **(G)** Number of cells per LSPCs colony after two weeks of culture in methylcellulose in the presence or absence of 100 pg/ml rhIL21 (two rounds of plating, n = 9). **(F, G)** Each dot represents the mean of three technical replicates. Different colors indicate different patients. Statistics were determined by Student's *t* test. Data are shown as mean ± SEM. **(H)** T cells cultured in vitro for 72 h in the presence or absence of 100 pg/ml rh-IL21 and stained for phospo-p38 as an internal control for the experiment shown in Fig. 5Q. Abbreviations: LSPCs, leukemic stem and progenitor cells; PB, peripheral blood; MFI, mean fluorescence intensity; HSPCs, hematopoietic stem and progenitor cells.

Figure S7

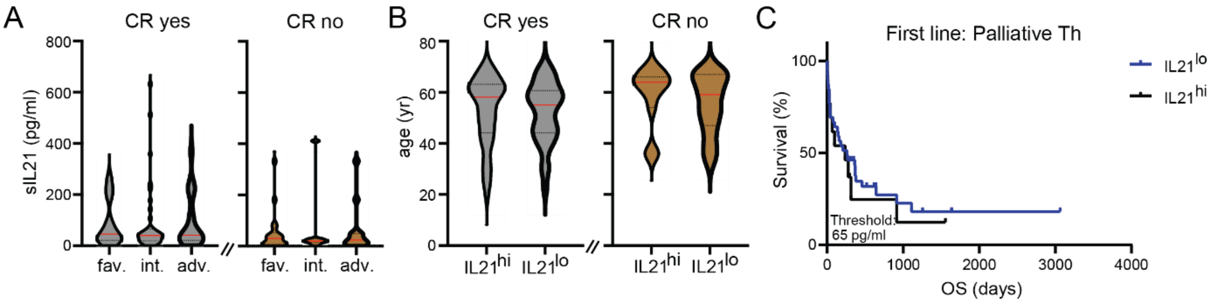

Figure S7. sIL21 levels are not influenced by risk group and age of patients undergoing intensive chemotherapy as first line therapy. sIL21 has no prognostic value for patients undergoing palliative first line therapy. Related to Figure 6. (A) sIL21 of patients that achieved and did not achieve CR, differentiated according to cytogenetic/molecular risk groups. Data are shown as mean ± SD. Statistics were determined by one-way ANOVA. **(B)** Age of patients that achieved and did not achieve CR differentiated according to sIL21 levels at the threshold of 35 pg/ml. Data are shown as mean ± SD. Statistics were determined by Mann-Whitney test. **(C)** Kaplan-Meier survival curves of the AML patients that received palliative first line therapy (n = 52) divided into two groups at the sIL21 threshold of 65 pg/ml. Statistics were determined by log-rank test. Abbreviations: CR, complete remission; fav., favorable; int., intermediate; adv., adverse; OS, overall survival; Th, therapy.

SUPPLEMENTARY TABLES

Table S2. Characteristics of AML patients analyzed by RNA-Seq.

Age at diagnosis, sex, risk category, percentage of PB blasts and BM infiltration, cytogenetic aberration and molecular diagnosis, immunophenotype and FAB are listed for each patient for which RNA-Seq was performed. Risk categories were determined based on the ENL 2022 guidelines[5].

Abbreviations: PB, peripheral blood, BM, bone marrow, adv., adverse; n.a., not available; MDS/MPS, myelodysplastic syndrome-myeloproliferative neoplasms.

**ID**	**Age at diagnosis**	**Sex**	**Risk**	**PB blasts (%)**	**BM infiltr. (%)**	**Cytogenetics**	**Molecular diagnosis**	**Immuno-phenotype**	**FAB**
1	54	f	adv.	85	90	Deletion 7; EVI-1 rearrangement	EVI-1 positive: NRAS mutated	CD34, CD38, CD117, HLA-DR, CD13, CD33, CD4, CD7 (partial) and CD56 (partial).	secondary AML therapy related
2	72	m	n.a.	20	n.a.	n.a.	n.a.	two blast subpopulation: one with CD34, HLA-DR, CD33, CD71 and one with CD34, CD33, CD11b, CD35 and CD71.	secondary AML from MDS/MPS
3	60	f	adv.	4.5	25	Deletion (20p)	SF3B1 and ASXL1 mutated	n.a.	AML-M2
